# Pangenome-driven discovery and comparative genomics of glycosyltransferase genes in *Camellia sinensis*

**DOI:** 10.3389/fpls.2026.1763078

**Published:** 2026-02-24

**Authors:** Jiuju Luo, Jing Liu, Xiaohuan Li, Zirong Li, Siwen Wu, Ligui Xiong, Haojing Shao

**Affiliations:** 1 Key Laboratory of Tea Science of Ministry of Education Hunan Agricultural University, Changsha, China; 2 Guangdong Laboratory for Lingnan Modern Agriculture, Genome Analysis Laboratory of the Ministry of Agriculture and Rural Affairs, Agricultural Genomics Institute at Shenzhen, Chinese Academy of Agricultural Sciences, Shenzhen, China; 3 National Research Center of Engineering and Technology for Utilization of Botanical Functional Ingredients, Changsha, China; 4 Yuelushan Laboratory, Changsha, China; 5 National Key Laboratory for Tea Plant Germplasm Innovation and Resource Utilization, Changsha, China

**Keywords:** *Camellia sinensis*, pan-gene family, pan-genome, structural variation, UGT

## Abstract

The quality of tea plants is determined by the accumulation of secondary metabolites including tea polyphenols, flavonoids, theanine and terpenoids. UDP-glycosyltransferase (UGT) genes catalyze the glycosylation of UDP-sugar donors to small-molecule acceptors, which directly modulates the structure, stability and biological activity of these quality-related metabolites. We systematically identified UGT gene family members across 22 high-quality tea plant pan-genomes. Evolutionary characteristics were analyzed via duplication type, Ka/Ks ratio and structural variation (SV) analysis. The expression patterns of CsUGT genes were investigated using expression comparison and transcriptomic data under normal and abiotic stress conditions. A total of 3,210 UGT genes were identified and classified into 201 orthologous groups (OGs), including 9 core, 24 soft-core, 116 dispensable and 52 private OGs. Whole-genome duplication (WGD) dominated core and soft-core gene duplication, while non-core genes were mainly derived from transposed or proximal duplications. Fifteen CsUGT genes underwent positive selection, and most were under purifying selection. SVs significantly affected the expression, conserved domains and cis-elements of CsUGT22, CsUGT14 and CsUGT43. CsUGT genes were highly expressed in tea plant buds and young leaves; CsUGT29, CsUGT43 and CsUGT49 were markedly upregulated under drought and salt stresses. This study reports the first comprehensive pan-genomic analysis of the tea plant UGT gene family, which elucidates its evolutionary dynamics and adaptive functional diversification. These findings establish a fundamental framework for future molecular research and breeding applications of CsUGT genes in tea plants.

## 1 Introduction

Tea plant [*Camellia sinensis* (L.) O. Kuntze], a major global beverage crop, is vulnerable to abiotic stresses such as low temperature and drought (Jin et al., 2023). Flavonoids, core secondary metabolites of tea plants, determine tea quality (color, aroma, taste) and regulate stress responses, with their contents and compositions varying by genetic background and environment (Liu et al., 2018). Cultivated tea plants (*Camellia* L., Sect. Thea (L.) Dyer) mainly include two core varieties: *Camellia sinensis* var. *assamica* (CSA) and *Camellia sinensis* var. *sinensis* (CSS), differing in morphology, stress tolerance, flavonoid profiles, and suitable cultivation areas; the rare transitional variety *Camellia sinensis* var. *pubilimba* (CSP) is intermediate and distributed in mountainous areas with complex climates (Kaundun and Matsumoto, 2003; Wei et al., 2018; Wu et al., 2022; Tong et al., 2025b; Wang et al., 2025). CSS accounts for approximately 67% of Chinese tea germplasm and plays a key role in yield improvement, quality optimization (via flavonoid regulation), and stress-resistant stable production (Wang et al., 2025). Gene family variation and conserved evolution provide insights into plant evolution and adaptation and flavonoid biosynthesis mechanisms (Hanada et al., 2008; Robertson et al., 2017; Yang et al., 2024), but current studies are limited to single-variety genomes. Systemic research on inter-varietal gene family differences and their associations with flavonoid metabolism and stress resistance is scarce, hindering comprehensive understanding of the molecular mechanisms underlying tea plant adaptive evolution and quality formation.

The UGT superfamily is the largest glycosyltransferase gene family in the plant kingdom, and is widely distributed across organisms ranging from structurally simple unicellular algae to mosses, ferns, and higher plants (Rehman et al., 2018; Song et al., 2019). Previous studies have investigated the UGT family in various crops, with the model plant *Arabidopsis thaliana* being the first to be studied, in which 122 UGT members have been identified and classified into 14 groups based on sequence homology (Zhang et al., 2021a). Currently, a large number of UGT gene members have also been identified in other crops, with 254, 212, and 261 members identified in apple, soybean, and upland cotton, respectively (Le Roy et al., 2016; Yin et al., 2017; Xiao et al., 2019). Several UGT genes have been functionally characterized in tea plants. For example, *UGT85A53*-catalyzed (Z)-3-hexenol glycosylation enhances tea plant (C. sinensis) resistance to *Ectropis obliqua* (Jing et al., 2020); and *UGT91Q2*-mediated nerolidol glycosylation improves cold tolerance (Zhao et al., 2020). Recently, *CsUGT75L12* and *CsUGT79B28* synergistically mediate flavanone 7-O-neohesperidoside biosynthesis via stepwise catalysis, clarifying the sequential glycosylation pathway of flavonoid diglycosides and the key molecular steps underlying the formation of tea plant bitterness traits (Dai et al., 2022). Additionally, *CsUGT78A15* specifically catalyzes flavonol 3-O-glucoside and 3-O-galactoside synthesis, with Gln373 (Q373) as the key active residue, thereby elucidating the molecular basis of flavonol glycoside structural diversity and its regulation of tea leaf coloration and antioxidant capacity (Cui et al., 2016).

Most existing studies on UDP-glycosyltransferase (UGT) genes in tea plants are limited to individual gene functional validation, lacking systematic comparisons across different tea plant varieties. This hinders comprehensive elucidation of the UGT family’s evolutionary patterns and its regulatory mechanisms linked to tea plant adaptability and quality traits.;To address this gap, we leveraged genomic resources from 22 representative varieties of the tea plant pan-genome (Chen et al., 2023). For the first time at the pan-genomic level, we integrated orthogroup identification, phylogenetic analysis, structural variation and transcriptomic profiling to systematically dissect the UGT family’s evolutionary dynamics and characteristic divergences across different tea plant varieties.; Tao et al.’s work on the extended tea plant pan-genome, which verified structural variation-driven trait divergence, corroborated and refined previous findings, thereby providing critical theoretical and empirical support for our study (Tao et al., 2025). Our results establish a novel theoretical framework for deciphering the UGT family’s evolutionary mechanisms and their regulatory roles in flavonoid biosynthesis and stress responses, while offering candidate gene resources and a molecular basis for tea plant stress-tolerant breeding and quality improvement.

## 2 Materials and methods

### 2.1 Identification of the UGT gene family in tea plants

To identify UGT family genes in the genomes of tea plant varieties, genomic data of 22 tea plant varieties (CSS, CSA, and CSP) were obtained from the study by Chen et al. (2023). A Hidden Markov Model (HMM) profile corresponding to the UDPGT domain (PF00201) was retrieved from the Pfam database (https://pfam.xfam.org/). This HMM profile was used with HMMER software (v3.3.2) to search the tea plant protein database, with an E-value cutoff of 1e-5. Additionally, 114 UGT protein sequences from *Arabidopsis thaliana* (downloaded from the TAIR database, https://www.arabidopsis.org/) were used as query sequences for BLAST analysis. A candidate UGT gene set was constructed via HMMER screening, followed by BLAST sequence alignment with a sequence identity threshold set at > 50% to obtain candidate UGT genes in tea plants (Ross et al., 2001). Subsequently, the Conserved Domain Database (CDD; https://www.ncbi.nlm.nih.gov/cdd/) was used to analyze and verify the conserved domains of the candidate proteins (Yang et al., 2023). Only sequences encoding proteins with the conserved UDPGT domain were retained, thus defining the final repertoire of UGT family genes across the 22 tea plant accessions.

### 2.2 Classification of core and non-core genes in the tea plant UGT gene family

We used OrthoFinder (v2.5.4) to cluster orthologous gene groups (OGs) of 22 tea plant pan-genomes and generate an OG list (Emms and Kelly, 2019). With BLAST as the sequence similarity search tool and OrthoFinder’s default parameters, MAFFT was used for multiple sequence alignment (MSA) of OGs, and FastTree for gene tree inference. Subsequently, based on the identified CsUGT genes, we obtained the corresponding OGs list for the CsUGT gene family. These CsUGT genes were classified into four categories: core genes (present in all 22 genomes), soft-core genes (present in ≥90% of the 22 genomes), dispensable genes (present in 2%–90% of the 22 genomes), and private genes (present in only one of the 22 genomes) (Tong et al., 2025a).

### 2.3 Phylogenetic analysis of the CsUGT gene family

Phylogenetic analysis was conducted using the protein sequences of UGT genes from *Arabidopsis thaliana* and tea plants. First, MUSCLE (v5.3) was used for multiple sequence alignment, followed by trimming with trimAl (v1.5.0). Subsequently, the phylogenetic tree was constructed via IQ-TREE (v2.1.4) using the Maximum Likelihood (ML) method with automatic optimal pruning, and branch support was assessed by 1000 ultrafast bootstrap replicates. Tree visualization was performed online with iTOL (https://itol.embl.de/) (Letunic and Bork, 2021).

### 2.4 Presence and absence analysis of CsUGT genes

Orthologous groups (OGs) were identified from the 22 tea plant pan-genomes using OrthoFinder (v2.5.4). Based on this, a presence/absence profile of each CsUGT gene across the 22 tea plant varieties was generated using the R package ComplexHeatmap.

### 2.5 Synteny analysis of CsUGT genes and identification of gene duplication types

The gene annotation files and coding sequences (CDS) downloaded from the study by Chen et al. (2023) were used to perform synteny analysis of 22 tea plant varieties based on JCVI (Tang et al., 2024).

Camellia oleifera (oil tea) data downloaded from the Tea Plant Information Archive (TPIA; https://tpia.teaplants.cn/download.html) was used as the outgroup. For comparative purposes, we merged the CsUGT family protein sequences from the 22 tea plant cultivars into a single FASTA file, followed by an all-vs-all BLASTp search against this file to evaluate sequence similarity. Based on gene IDs, we extracted the coordinates of CsUGT genes from the GFF3 annotation file of each cultivar and merged these coordinates into a BED-formatted file to standardize gene positions across germplasms. We further constructed a database using the UGT protein sequences identified from C. oleifera. After processing the C. oleifera data as described above for tea plants, we applied the DupGen_finder-unique.pl script of DupGen_finder to determine the duplication types of UGT genes, including whole-genome duplication (WGD), tandem duplication (TD), proximal duplication (PD), transposed duplication (TRD), and dispersed duplication (DSD) (Qiao et al., 2019).

### 2.6 Ka/Ks calculation

The coding sequences (CDS) and protein sequences of CsUGT family members were retrieved from 22 tea genomes, and the Ka/Ks values for each pair of homologous UGT genes were calculated using Ka/Ks Calculator software (Wang et al., 2010); the R packages ggridges and ggplot2 were employed to generate the ridgeline plot of Ka/Ks values.

### 2.7 Construction of the pan-genome map, and expression analysis of CsUGT genes overlapping with gene annotations and structural variations

Structural variation (SV) data and third-generation sequencing data were obtained from the study by Chen et al. (2023). VG (v1.61.0) was used to reconstruct the tea plant pan-genome map, and SV variation profiles of 22 tea plant accessions were acquired (Garrison et al., 2018). Subsequently, Bcftools (v1.13) was employed for merging and filtering with the parameter set to DP ≥ 20. Python scripts were utilized to identify variant types, and SnpEff (v5.2) was applied for gene annotation analysis using the genome of cultivar TGY as the reference genome (Cingolani et al., 2012).

Using the aforementioned approach, we obtained SV locus information for each tea plant cultivar. Among the gene expression data of these cultivars, data for 17 were obtained from Chen et al. (2023); while data for cultivars HD, LJ43, TGY, and SCZ were obtained from Wang et al. (2021), Wang et al. (2020), Zhang et al. (2021b), and Xia et al. (2020), respectively. After undergoing conversion, filtering, alignment, quantification, and merging, the expression level list of buds and young leaves from 21 different tea plant cultivars was obtained (excluding QL10H). In-house Perl scripts were used to assess whether CsUGT genes overlapped with SVs in each variant. If a CsUGT gene was found to overlap with an SV, its expression data in the corresponding cultivar was classified as SV-associated gene expression data; otherwise, it was categorized as SV-absent gene expression data. Pearson correlation coefficients between the presence of SVs overlapping with genes and gene expression levels were calculated. CsUGT genes with p < 0.05 were considered to exhibit significant changes in expression levels due to SVs.

### 2.8 Analysis of the gene structure, conserved motifs, and cis-acting elements of CsUGT genes under the influence of structural variations

Conserved motifs were identified using MEME program (http://meme-suite.org/) with the number of motifs set to 10; gene structures were analyzed based on gene annotation files (GFF3). Subsequently, Chiplot and CFVisual were respectively used for visualization.

Additionally, TBtools was used to extract the 2000-bp promoter sequences of cultivar HD (the cultivar with the most overlaps with structural variations, SVs) and the reference genome TGY. Subsequently, online analysis was conducted using the PlantCARE database (https://bioinformatics.psb.ugent.be/webtools/plantcare/html/), followed finally by visualization with R (Chen et al., 2020).

### 2.9 RNA-seq data analysis

Transcriptome data of TGY under drought stress and salt stress were obtained from the study by Zhang Qing et al., and transcriptome data of roots, stems, leaves, flowers, and buds were obtained from the study by Zhang et al. (2017, 2021b). Low-quality sequences were removed using fastp (Chen et al., 2018), and STAR was used for mapping clean reads to the reference genome sequence. Subsequently, featureCounts (from the Subread package) was used for read counting. Finally, custom Python scripts were used to calculate TPM values, which were visualized using R.

## 3 Result

### 3.1 Homologous gene list of the tea plant pan-genome and identification of CsUGT genes

**Figure 1:**
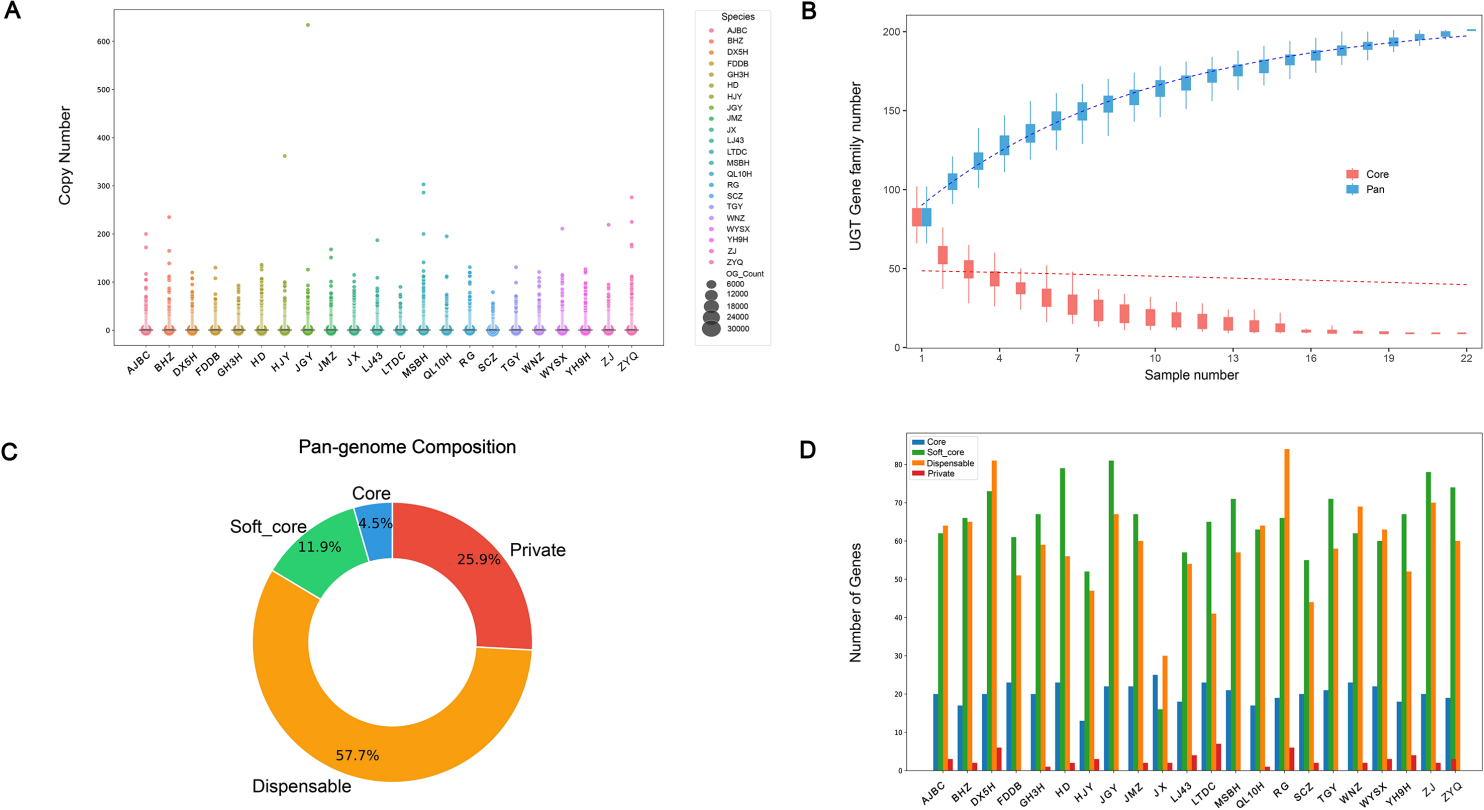
Overview of the tea plant pan-genome based on homology **(A)** Number and distribution of orthologous gene clusters (OGs) among different tea plant varieties; **(B)** Variation patterns of pan-genome (red) and core gene (blue) numbers with the increase in the number of sampled genomes. **(C)** Proportion distribution of core OGs, soft-core OGs, dispensable OGs, and private OGs in the UGT gene family within the pan-genome. **(D)** Distribution numbers of core genes, soft-core genes, dispensable genes, and private genes of the UGT gene family among 22 different tea plant varieties.

OrthoFinder (v2.5.4) was used to identify orthologous groups (OGs) from the pan-genomes of 22 tea plant cultivars, yielding in a total of 56,715 OGs. The copy number of most OGs across the 22 cultivars ranged from 0 to 200, indicating relative conservation. However, cultivars BHZ, HJY, JGY, MSBH, WYSX, ZJ, and ZYQ had more than 200 copies of certain OGs, suggesting that these cultivars may have experienced specific gene family expansion events (**Figure 1A**). In the pan-genome analysis, the total number of CsUGT genes increased with the number of genomes, while the number of core CsUGT genes decreased and tended to stabilize (**Figure 1B**). A combination of HMMER search and BLASTp program was used for UGT family member identification, leading to the discovery of 3,210 CsUGT genes in the tea plant pan-genome (**Supplementary Figure S1**). Based on the homologous gene list, these genes were clustered into 201 orthologous groups (OGs) (**Supplementary Table S1**). Of these, 9 were core OGs (present in all 22 cultivars), 24 were soft-core OGs (present in 20–21 cultivars), 116 were dispensable OGs (present in 2–19 cultivars), and 52 were private OGs (present in only one cultivar). Among these OGs, dispensable OGs accounted for the largest proportion (57.7%) of the entire CsUGT family, while core OGs accounted for the smallest (4.5%) (**Figure 1C**). Except for cultivars FDDB, JGY, MSBH, and TGY, all other tea plant cultivars contained all four types of CsUGT OGs. Specifically, JX had the highest number of core genes (25 in total), JGY had the most soft-core genes (81), RG the largest number of dispensable genes (84), and LTDC had the most private genes (7 in total) (**Figure 1D**).

### 3.2 Phylogeny, presence-absence variation, and CNV of CsUGT genes in tea plants

**Figure 2:**
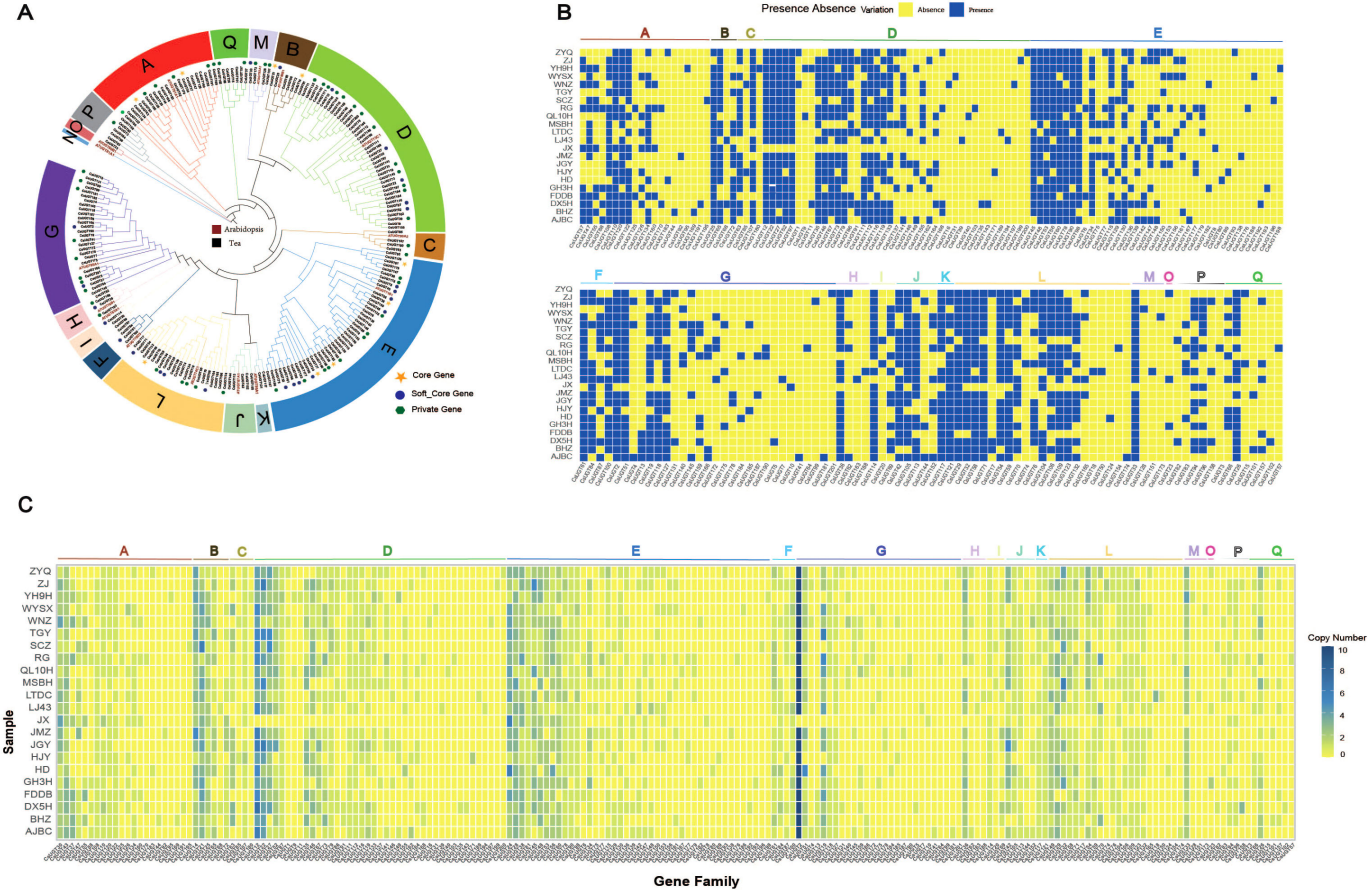
Phylogenetic analysis, PAV, and CNV of CsUGT genes **(A)** Phylogenetic tree of UGT genes from Arabidopsis thaliana and tea plants. **(B)** Heatmap showing the presence and absence of 192 UGT genes in 22 tea plant varieties, excluding core genes. **(C)** Heatmap of CNV of UGT genes in the tea plant pan-genome.

To further investigate the evolutionary relationships among UGT gene families of different tea plant cultivars, a phylogenetic tree was constructed using UGT protein sequences from tea plants and *Arabidopsis thaliana*. Based on the AtUGT classification system (Li et al., 2014), CsUGTs and AtUGTs can be divided into 17 groups (Group A to Group Q) (Rehman et al., 2018). CsUGT genes are mainly distributed in Groups A–M and O–Q, with no CsUGT genes detected in Group N. Among these groups, Group E (43) and Group D (41) contain the largest number of CsUGT genes, while Group O (1) contains the smallest. This indicates distinct differences in the distribution of CsUGT genes across groups (**Figure 2A**). **Figure 2B** shows the presence/absence of CsUGT genes (excluding core OGs) among the 22 tea plant cultivars. In the phylogenetic tree, except for Groups B, F, I, J, K, N, and O—where no cultivar-specific private CsUGT genes were found—all other groups contain private genes. This suggests that the private genes of certain tea plant cultivars in these other groups may be associated with the unique traits of the respective cultivars. To evaluate the copy number variation (CNV) of the UGT gene family in the tea plant pan-genome, we calculated the gene copy number for each pan-genome. Overall, the number of CsUGT genes in each pan-genome ranges from 1 (a unique UGT in specific tea plant cultivars) to 34 (present in multiple copies in the genomes of certain tea plants). Among the 201 CsUGT OGs, for CsUGT2, the copy number in all tea plant cultivars reached more than 20, except for JX where the copy number was 0. For CsUGT24, JX had the highest copy number (6). This gene (CsUGT24) existed in 2–3 copies in some genomes, but it was absent in others. These multi-copy genes may represent UGTs under active replication and play an important role in microenvironmental adaptation (**Figure 2C**).

### 3.3 Synteny of CsUGT genes and distribution of different duplicated gene types

**Figure 3:**
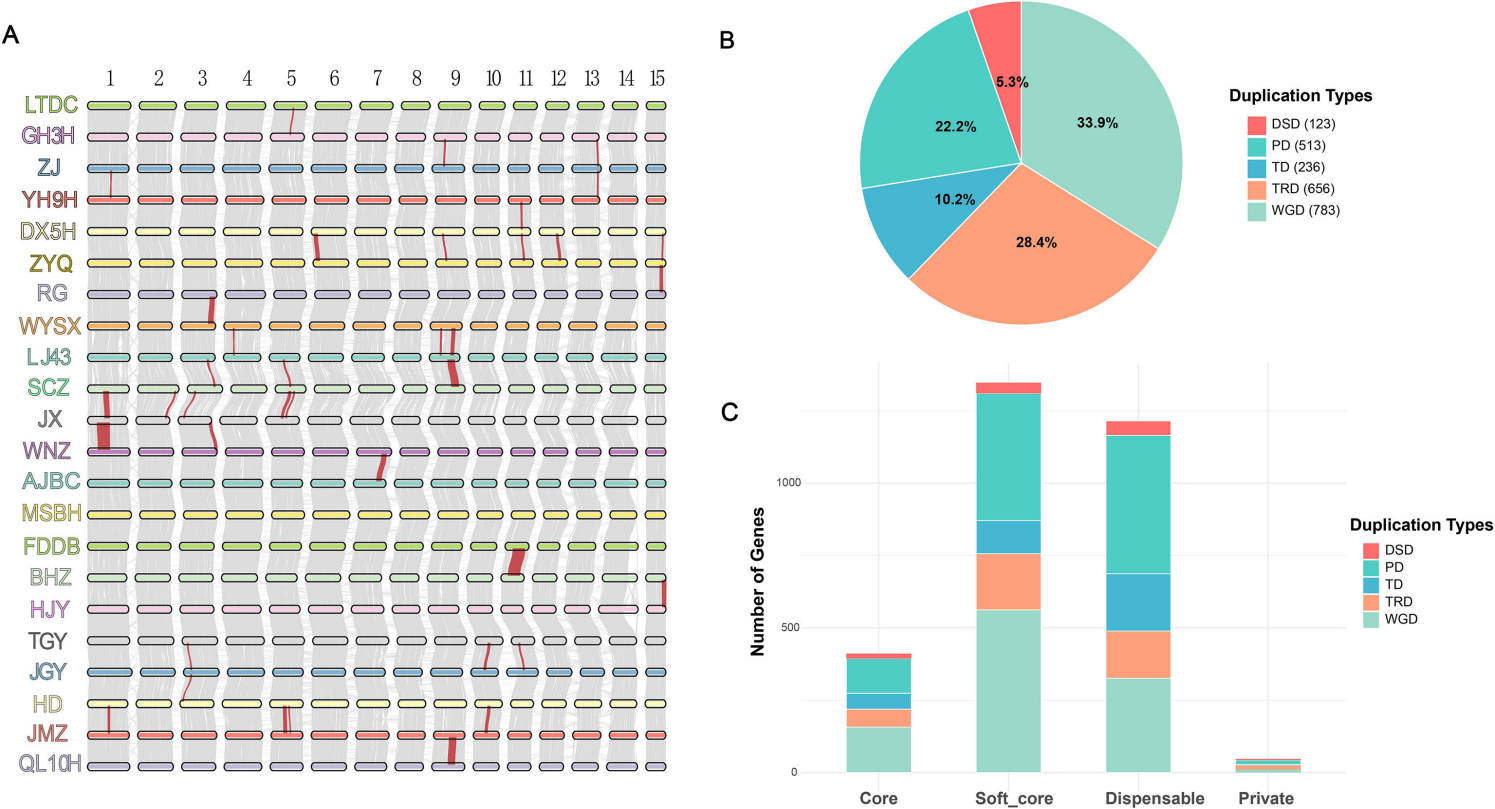
Synteny and gene duplication types of UGT genes in 22 tea plant varieties **(A)** Synteny of UGT genes on 15 chromosomes among 22 different tea plant varieties, with red lines representing syntenic CsUGT gene pairs. **(B)** Pie chart of CsUGT gene duplication types. **(C)** Stacked bar chart of CsUGT gene duplication types classified by category.

Based on the evolutionary relationships of 22 tea plant cultivars reported by Chen et al. (2023), collinearity analysis of the UGT gene family across different cultivars revealed that significant collinear blocks were detected between all other cultivar pairs, except for the absence of significant collinear blocks between AJBC and MSBH, MSBH and FDDB, and HJY and TGY. Notably, relatively thick collinear blocks were observed between JX and WNZ, and between FDDB and BHZ, indicating high conservation of UGT genes among these cultivars during evolution (**Figure 3A**).

To further explore the evolutionary dynamics of CsUGT genes, we identified their duplication types. Following the priority order of duplicated genes (WGD > TD > PD > TRD > DSD), a total of 2,311 gene duplication pairs with different patterns were identified. Among these, whole-genome duplication (WGD) accounted for the largest proportion (33.9%, 783 pairs), followed by transposed duplication (TRD, 28.4%, 656 pairs), proximal duplication (PD, 22.2%, 513 pairs), tandem duplication (TD, 10.2%, 236 pairs), and dispersed duplication (DSD) accounting for the smallest proportion (5.3%, 123 pairs) (**Figure 3B**). For core and soft-core genes, WGD was the dominant duplication type, with 157 and 562 gene pairs, respectively. In contrast, proximal duplication (PD) was the most abundant type in dispensable genes (478 pairs). Private genes were mainly characterized by proximal duplication (PD, 15 pairs) and transposed duplication (TRD, 13 pairs) (**Figure 3C**). Further analysis of duplication patterns across different CsUGT categories revealed distinct distribution characteristics, indicating that WGD is the primary evolutionary driving force underlying the expansion, functional differentiation, and environmental adaptation of the CsUGT gene family.

### 3.4 CsUGT genes undergo different selection pressures among tea plant varieties

**Figure 4:**
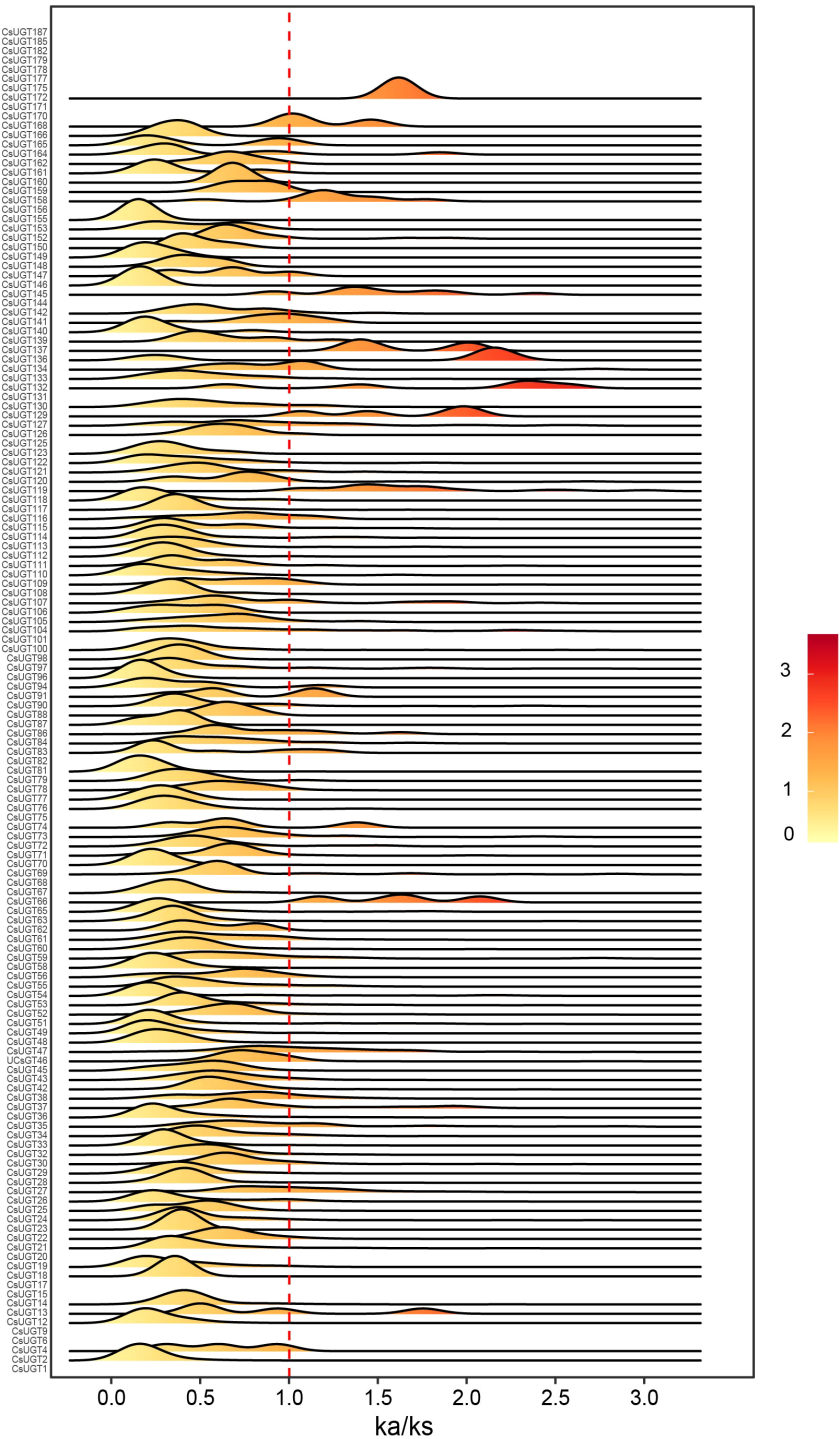
Distribution of Ka/Ks values of CsUGT genes in 22 tea plant varieties.

Analysis of the Ka/Ks value can reveal the selection pressure acting on gene family members during the formation of different varieties. To explore the selection pressure on CsUGT genes, we calculated the Ka/Ks value for each CsUGT gene based on the gene sequences from the pan-genome of 22 tea plant varieties (**Figure 4**).

Since CsUGT190, CsUGT187, CsUGT182—CsUGT185, CsUGT178, CsUGT171, CsUGT170, CsUGT167, CsUGT131, CsUGT125, CsUGT101, CsUGT85, CsUGT82, CsUGT68, CsUGT20, CsUGT15, CsUGT11, CsUGT9, CsUGT6, and CsUGT1 are present in only two varieties, they can only generate one Ka/Ks value. Although CsUGT179, CsUGT177, CsUGT175, CsUGT156, CsUGT144, CsUGT75, and CsUGT17 exist in three varieties, they have NA values. The remaining genes that do not appear are present in only one variety. Most CsUGT genes have Ka/Ks values ranging from 0 to 1, though the positions of their peaks differ. Among the 22 tea plant pan-genomes, it is evident that CsUGT13, CsUGT66, CsUGT74, CsUGT91, CsUGT119, CsUGT129, CsUGT132, CsUGT134, CsUGT136, CsUGT137, CsUGT141, CsUGT145, CsUGT158, CsUGT168, CsUGT168, CsUGT172, and others exhibit Ka/Ks values greater than 1. This indicates that these genes were under positive selection pressure during tea plant domestication.

### 3.5 The expression, structure, and promoter cis-elements of the CsUGT gene are affected by structural variations

**Figure 5:**
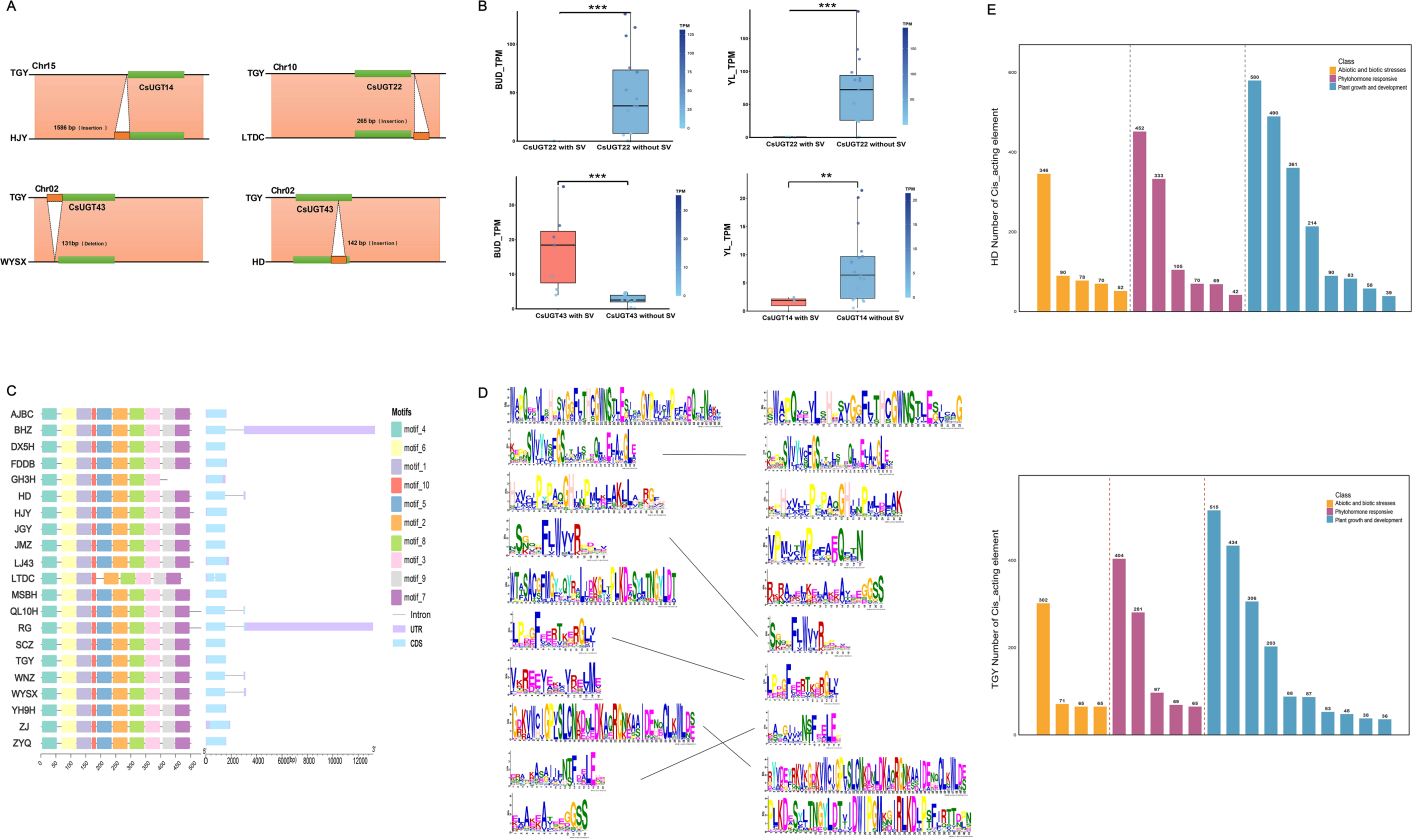
shows structural variations (SVs) affecting gene expression, structure, motifs, and promoter cis-acting elements in the pan-genomes of different tea plant varieties: **(A)** Showing insertions or deletions of structural variations (SVs) within the 2-kb regions upstream and downstream of the CsUGT14, CsUGT22, and CsUGT43 genes. **(B)** SVs significantly affected the expression of CsUGT22 and CsUGT43 in buds, as well as CsUGT22 and CsUGT14 in young leaves (***P < 0.001, **P < 0.01). **(C)** Schematic diagram of the gene structure and motifs of CsUGT22 across 21 tea plant varieties (excluding JX). **(D)** The left and right panels display the Weblogo plots of CsUGTs from HD and the reference genome, respectively, with Weblogo plots connected by lines indicating mutual correspondence and all plots arranged in descending order of E-values. **(E)** Statistical analysis of the number of cis-acting elements in the promoters of CsUGTs from HD and the reference genome (TGY).

First, variation data of UGT family members were extracted from tea plant structural variation files. Subsequently, expression level data of young leaves and buds were selected, and Pearson correlation coefficients of gene expression were calculated between SV-overlapping and non-overlapping UGT genes. Compared with the reference genome, the main types of structural variations (SVs) were deletions and insertions, overlapping with the coding regions of CsUGT genes and their flanking 2-kb upstream and downstream sequences ((**Figure 5A**). Subsequently, we analyzed the expression correlation between SV-overlapping and non-overlapping genes. CsUGT14, CsUGT22, and CsUGT43 showed significant expression differences between the two groups in buds and young leaves of 21 tea plant varieties (excluding QL10H), indicating that structural variations (SVs) significantly affect the expression of these three genes (**Figure 5B**). In gene expression analysis, CsUGT22 was the gene showing significant differences in both tissues. Thus, we characterized its gene structure and conserved domains across 21 tea cultivars (excluding JX). The domain of CsUGT22 in most varieties’ pan-genomes matched the reference genome (TGY), but notable variations were detected in GH3H and LTDC. Moreover, seven cultivars (BHZ, HD, LTDC, QL10H, RG, WNZ, WYSX) carried two exons in CsUGT22, potentially altering its function (**Figure 5C**).

We quantified SV-overlapping genes between the reference genome TGY and other genomes, then selected the cultivar HD with the highest overlap to generate a conserved domain alignment plot (**Figure 5D**). In HD’s pan-genome, 5 conserved domains matched TGY while the other 5 were misaligned; incomplete amino acid alignment in corresponding domains indicated that SVs significantly affect CsUGT conserved domains. We further analyzed promoter cis-acting elements, selecting the top 20 most abundant elements for statistical analysis and visualization. TGY and HD differed in cis-acting elements related to plant growth, phytohormone response, and biotic/abiotic stress (**Supplementary Table S2**): HD had 8, 6, and 5 elements for these processes, respectively, whereas TGY had 10, 5, and 4 (**Figure 5E**). Collectively, SVs altered cis-acting element composition in tea UGTs, potentially affecting physiological processes like photosynthesis and hormone responses.

### 3.6 Atypical UGT genes are widely expressed in tea plants

**Figure 6:**
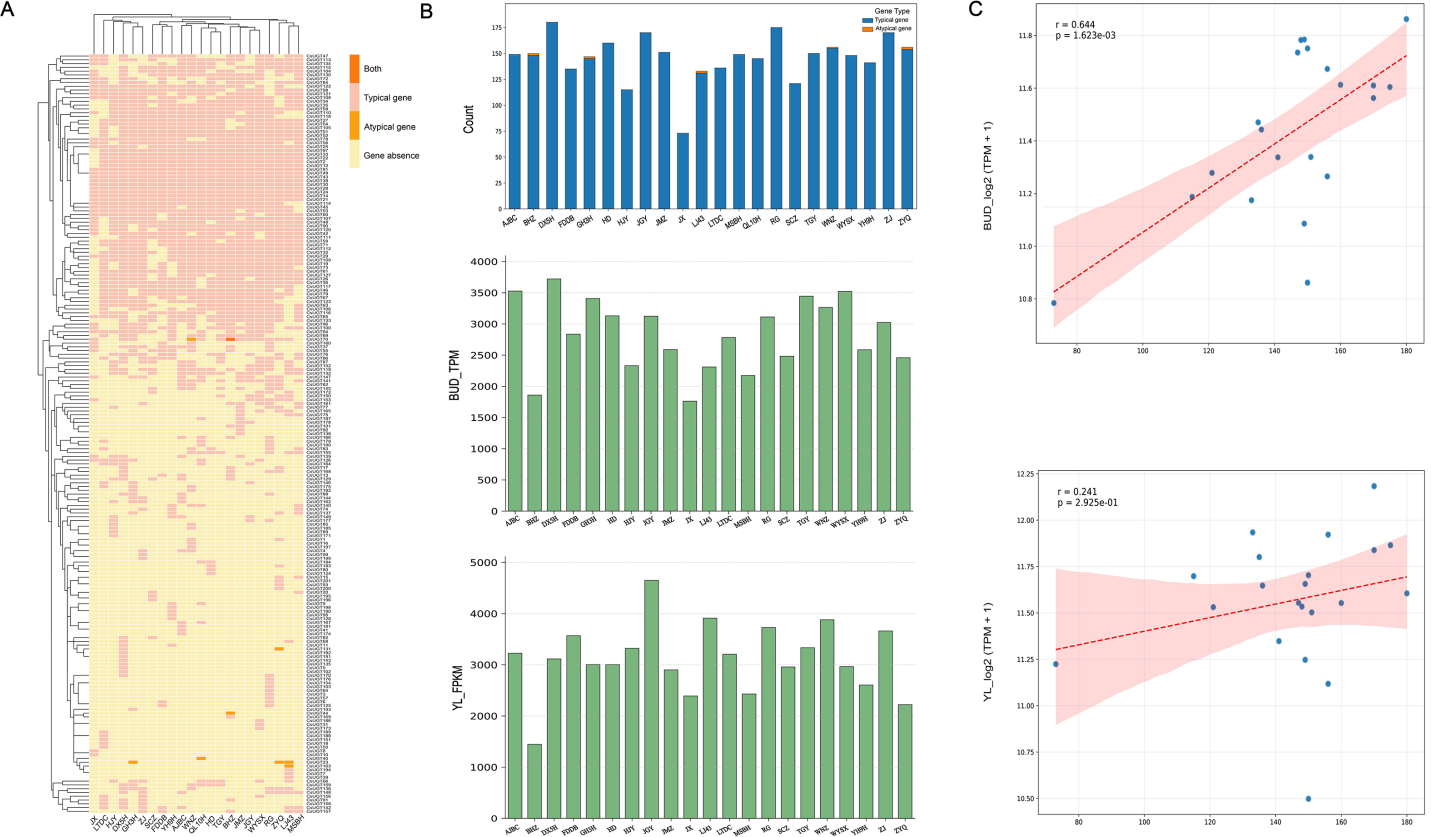
Widespread expression of typical and atypical CsUGT genes **(A)** Heatmap showing the classification of each CsUGT gene as typical (containing the PSPG motif) or atypical (not containing the PSPG motif) across different tea plant varieties. **(B)** Total number of UGT genes in the pan-genomes of different tea plant varieties and their total expression dosage in buds and young leaves. **(C)** Schematic diagram of Pearson correlation analysis: the upper right panel shows the correlation between buds and the total number of UGT genes across different varieties, while the lower right panel shows the correlation between young leaves and the total number of UGT genes across different varieties.

Since the identification of the gene family was based on conserved domain searching, we quantified the number of typical (containing the PSPG motif) and atypical (not containing the PSPG motif) UGTs in the pan-genomes of 22 tea plant varieties to further investigate the impact of SVs on the CsUGT gene family. Most genes were typical CsUGTs, while CsUGT163, CsUGT23, CsUGT40, and CsUGT44 were atypical CsUGTs. The remaining two genes, CsUGT131 and CsUGT70, exhibited both types (**Figure 6A**). To determine whether there is a relationship between the number of CsUGT genes and their total expression levels among different varieties, we counted the number of CsUGT genes and their total expression levels in buds and young leaves of each variety (excluding QL10H). There were certain differences in the number of CsUGT genes and their total expression levels across varieties: the number of CsUGT genes in different varieties ranged from 73 to 180. For buds, the highest TPM value among different varieties was 3721.4 (DX5H), and the lowest was 1762.5 (JX); for young leaves, the highest TPM value among different varieties was 4650.8 (JGY), and the lowest was 1445.3 (BHZ) (**Figure 6B**). A Pearson correlation analysis was performed to examine the relationship between the total number of CsUGT genes and the CsUGT log_2_TPM values in tea plant buds and young leaves. The results showed that for tea plant buds, the correlation coefficient (r) and significance test p-value between the total number of CsUGT genes and CsUGT log_2_TPM were 0.644 and 0.001623, respectively, indicating a significant correlation. For young tea plant leaves, the correlation coefficient (r) and significance test p-value between the total number of CsUGT genes and CsUGT log_2_TPM were 0.241 and 0.2925, respectively, showing no correlation (**Figure 6C**). These findings indicate that different tea plant tissues exert distinct effects on the relationship between these genes and their total expression levels.

### 3.7 Expression profiles of CsUGT genes under stress conditions based on RNA-seq data

**Figure 7:**
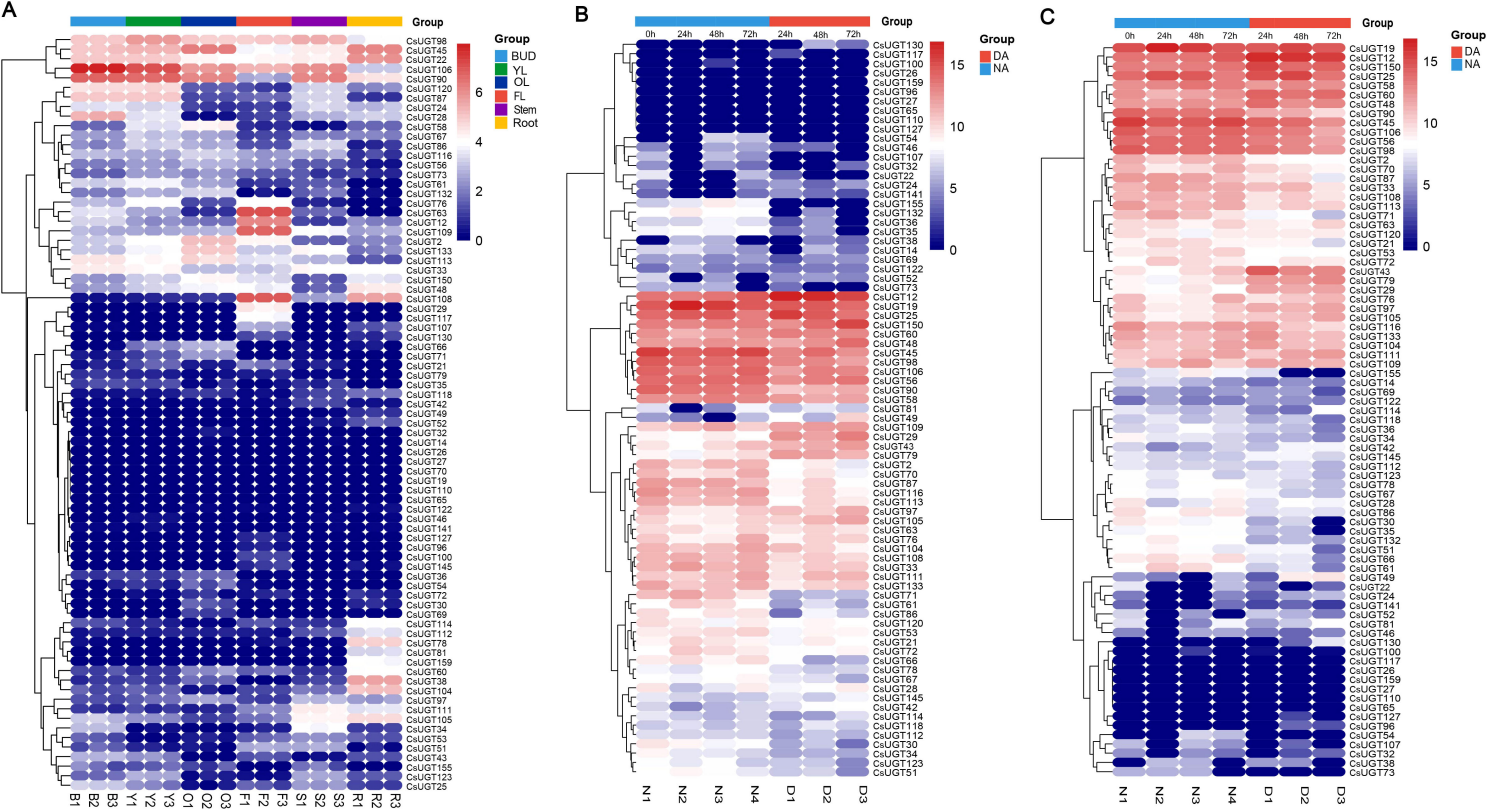
Expression profiles of the UGT gene family in different tissues and under stress conditions **(A)** Heatmap of CsUGT gene expression in different tissues; **(B)** Heatmap of CsUGT genes under drought stress; **(C)** Heatmap of CsUGT genes under salt stress.

To analyze the transcriptional profile characteristics of CsUGT genes, we examined transcriptome sequencing data (Zhang et al., 2021b), which covers six tissues in the tea plant genome (buds, young leaves, old leaves, flowers, stems, and roots). Additionally, to further investigate the response of CsUGT genes to drought and salt stresses, we obtained relevant data from the study by Zhang et al. (2017). The expression levels significantly differed among different tissues: most genes exhibited higher expression levels in buds and young leaves, while genes with significantly upregulated expression in roots included CsUGT45, CsUGT22, CsUGT78, CsUGT38, and CsUGT104 (**Figure 7A**). Under drought stress, CsUGT130, CsUGT49, CsUGT109, CsUGT29, and CsUGT43 showed upregulated expression levels at 24, 48, and 72 hours, respectively; additionally, the expression levels of CsUGT25 and CsUGT150 increased at 24 and 72 hours, respectively (**Figure 7B**). Under hydrochloric acid stress, several CsUGT genes displayed upregulated expression levels at 24, 48, and 72 hours, such as CsUGT12, CsUGT60, CsUGT43, CsUGT79, CsUGT29, CsUGT76, CsUGT97, CsUGT105, and CsUGT49 (**Figure 7C**). In conclusion, these results highlight the multifunctional roles of the CsUGT gene family in various stress response pathways and emphasize their importance in plant defense mechanisms.

## 4 Discussion

The tea plant pan-genome, constructed from 22 elite cultivars, represents the first comprehensive genetic resource capturing the species-wide diversity of tea plant. With this expanded genomic framework, researchers can now perform more extensive and integrated analyses of gene families across tea cultivars. Compared with single-reference genome studies, the pan-genome provides a far more complete picture of the genetic diversity of tea plants, encompassing copy number variations (CNVs), allelic diversity, and cultivar-specific genes. This enables a better understanding of the evolutionary dynamics and functional diversification of the UGT gene family, which is known to regulate secondary metabolism and environmental adaptability.

Previous studies on the UGT family in *Arabidopsis thaliana* (Lin et al., 2016) and *Oryza sativa* (Dong et al., 2020) have revealed its extensive functional diversity. However, tea plants—an economically important crop rich in unique secondary metabolites such as catechins, theanine, and volatile terpenoids—have long lacked such systematic, multi-genome analyses. Our pan-genome approach, incorporating 22 high-quality genomes with TGY as the reference, thus provides the first opportunity to dissect UGT gene family evolution at the species-wide level.

Core evolutionary characteristics of the UDP-glycosyltransferase (UGT) gene family in Camellia sinensis: whole-genome duplication (WGD) and tandem duplication (TD) synergistically drive expansion, with specific amplification of subfamily G and significant contraction of subfamily H. Distinct from Arabidopsis thaliana and Oryza sativa, this feature results from combined natural selection and artificial domestication, supporting tea plant core quality traits (e.g., flavonoid metabolism, aroma) and stress adaptability.;Orthogroup (OG) analysis confirmed these characteristics: 201 CsUGT OGs were identified, with core OGs (only 9) accounting for the lowest proportion and non-essential/private OGs being more abundant. This composition ensures genomic complementarity among cultivars, underpinning variety-specific traits.

Duplication type distribution: core/near-core genes dominated by WGD (ensuring evolutionary stability, functional redundancy and stable key biological functions); non-essential/private genes rich in tandem duplication (TD) and proximal duplication (PD), implying potential roles in adaptive evolution. This aligns with the classic principle: “conserved genes maintain survival, variable genes drive adaptation and phenotypic diversification” (Jelesko et al., 1999; Flagel and Wendel, 2009; Kuzmin et al., 2022). Cross-species comparisons showed species-specific UGT evolution: Arabidopsis thaliana relies on segmental duplication (14 phylogenetic groups) (Li et al., 2001); Oryza sativa expands via WGD (unique subfamily P) (Shen et al., 2025); Vitis vinifera UGTs unevenly distributed across 17 groups (A-P, R), with the most in group E (Hou et al., 2025). These reflect plant UGT diversity and aid in interpreting tea plant UGT evolution.;Multi-species UGT subfamily comparisons revealed conserved functional differentiation (subfamily-specific metabolic pathway associations): polyphenol/flavonoid-related UGTs in A, E, F, L, M (Li et al., 2001; Frydman et al., 2004; Montefiori et al., 2011; Yonekura-Sakakibara et al., 2012; Frydman et al., 2013; Song et al., 2016; Su et al., 2017; Rehman et al., 2018); terpenoid-related in D, M (Rahimi et al., 2019; Zhao et al., 2020); hormone-related in H, L, K, O (Dong and Hwang, 2014; Yin et al., 2017; Rehman et al., 2018; Mateo-Bonmatí et al., 2021). Phylogenetic analysis predicts tea plant UGT functions: UGT22 (group D) may participate in terpenoid glycosylation (regulating aroma intensity/durability); UGT43 (group A) may involve in polyphenol/flavonoid glycosylation (reducing tea bitterness, improving storage stability). These conserved features support UGT function prediction.

Structural variations (SVs)—including deletions, insertions, CNVs, inversions, and translocations—represent another important mechanism shaping genome plasticity and phenotypic diversity (Gabur et al., 2019; Yuan et al., 2021). In this study, SVs significantly affected the expression of CsUGT22, CsUGT43, and CsUGT14, particularly CsUGT22, whose expression varied markedly between buds and young leaves. Similar to previous findings in peach and maize, where SVs altered key regulatory genes controlling organ development (Chen et al., 2021; Guan et al., 2021), these results suggest that structural variations may have contributed to the diversification of CsUGT-mediated metabolic regulation among tea cultivars. Furthermore, SVs also influenced the conserved domains and cis-regulatory elements of CsUGT genes, indicating potential alterations in their enzymatic activity and transcriptional responsiveness.

The sensory attributes of tea—its color, aroma, taste, and shape—are largely determined by tissue-specific gene expression. Differences in transcript levels across tissues reflect the spatial–temporal distribution of key quality-related metabolites, forming the molecular foundation for raw material selection and tea quality improvement (Lin et al., 2021). As a subtropical evergreen crop, tea plant thrives under moist, acidic conditions but is sensitive to drought and salinity. Drought stress disrupts water balance and physiological metabolism, while salt stress induces combined ionic, osmotic, and oxidative stresses that limit growth and yield (Wan et al., 2024; Zheng et al., 2025). Transcriptomic analyses revealed that CsUGT22 is broadly expressed across tissues and exhibits downregulation under drought and salt stresses, suggesting a fundamental housekeeping function essential for maintaining cellular homeostasis. Conversely, CsUGT43 shows strong expression in buds and young leaves and remains upregulated under abiotic stresses, implying its involvement in maintaining shoot growth and secondary metabolite synthesis during stress responses.

Despite extensive pan-genome studies in crops such as maize, barley, and cotton, research on the tea plant pan-genome remains limited. By integrating the genomes of CSS, CSA, and CSP cultivars, this study provides the first systematic and comparative analysis of the UGT gene family across diverse tea plant varieties. These findings not only deepen our understanding of the evolutionary and functional diversification of UGTs in tea plants but also lay the groundwork for future molecular breeding aimed at improving tea quality and stress resilience.

## Data Availability

The original contributions presented in the study are included in the article/**Supplementary Material**. Further inquiries can be directed to the corresponding authors.
