# Supplementary material for: Pangenome-driven discovery and comparative genomics of glycosyltransferase genes in Camellia sinensis
Source: Front Plant Sci. 2026 Feb 24;17:1763078. doi: 10.3389/fpls.2026.1763078 (PMC12971411; doi:10.3389/fpls.2026.1763078)
Supplement: Supplementary file 1 [file DataSheet1.docx]

**Supplementary Materials**


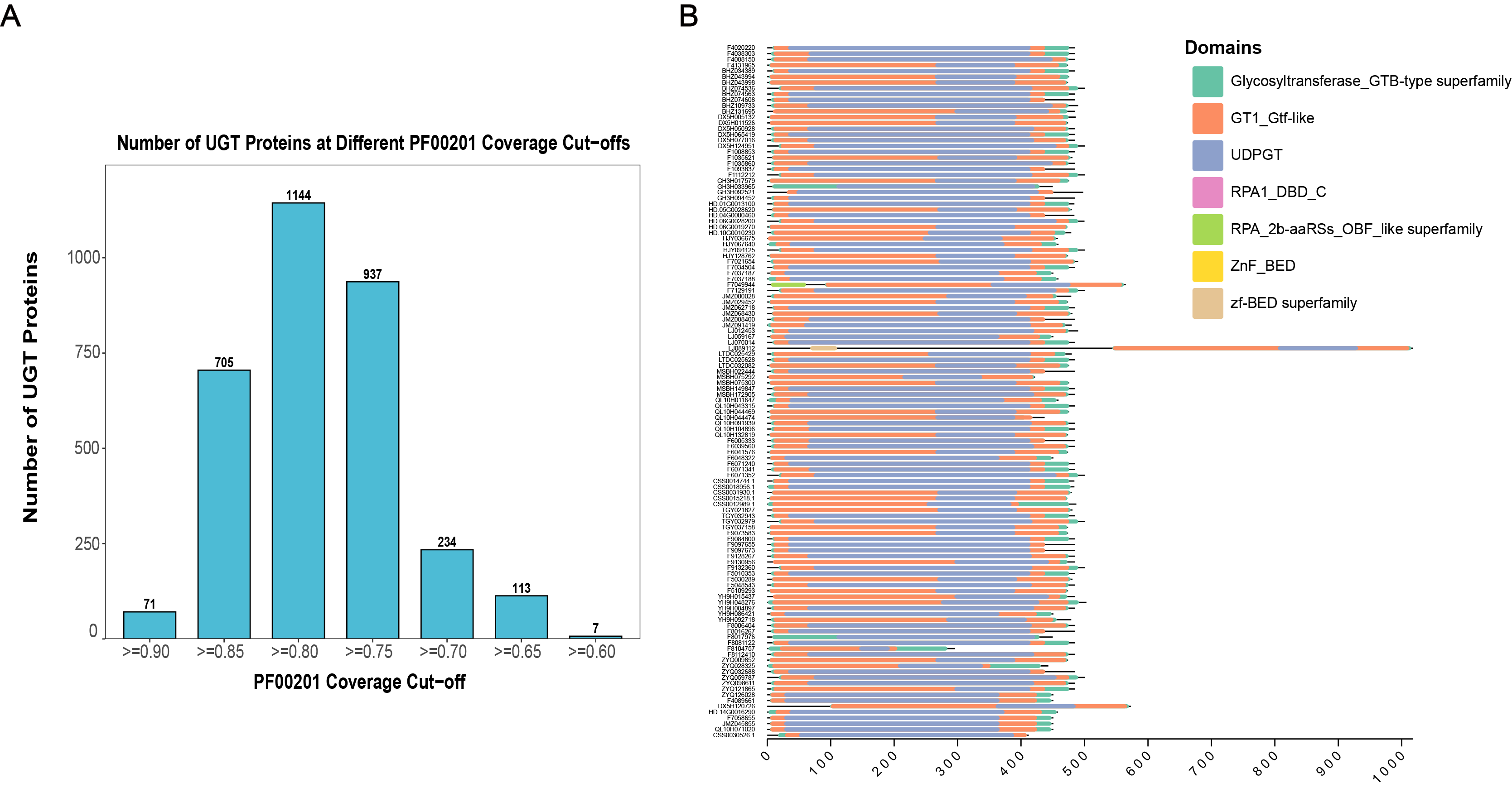


**Figure S1** PF00201 domain coverage thresholds of 3,210 CsUGT genes(A): Number of genes distributed across different percentage ranges of PF00201 domain coverage thresholds among 3,210 CsUGT genes. (B):Domain distribution of CsUGT genes with PF00201 domain coverage < 70%, all containing UDPGT.

| **Table S1** **201 orthologous groups (OGs) of the UGT gene family** | | | | | | | | | | | | | | | | | | | | | | |
| --- | --- | --- | --- | --- | --- | --- | --- | --- | --- | --- | --- | --- | --- | --- | --- | --- | --- | --- | --- | --- | --- | --- |
| **Fam_UGT** | **A**  **J**  **B**  **C** | **B**  **H**  **Z** | **D**  **X**  **5**  **H** | **F**  **D**  **D**  **B** | **G**  **H**  **3**  **H** | **H**  **D** | **H**  **J**  **Y** | **J**  **G**  **Y** | **J**  **M**  **Z** | **J**  **X** | **L**  **J**  **4**  **3** | **L**  **T**  **D**  **C** | **M**  **S**  **B**  **H** | **Q**  **L**  **10**  **H** | **R**  **G** | **S**  **C**  **Z** | **T**  **G**  **Y** | **W**  **N**  **Z** | **W**  **Y**  **S**  **X** | **Y**  **H**  **9**  **H** | **Z**  **J** | **Z**  **Y**  **Q** |
| **UGT1** | **0** | **0** | **0** | **0** | **0** | **0** | **0** | **0** | **0** | **0** | **0** | **0** | **0** | **0** | **0** | **0** | **0** | **1** | **0** | **0** | **0** | **1** |
| **UGT2** | **25** | **29** | **26** | **25** | **24** | **34** | **18** | **34** | **27** | **0** | **21** | **28** | **27** | **23** | **23** | **17** | **24** | **25** | **23** | **27** | **29** | **30** |
| **UGT3** | **0** | **0** | **0** | **0** | **0** | **0** | **0** | **0** | **0** | **0** | **0** | **0** | **0** | **0** | **1** | **0** | **0** | **0** | **0** | **0** | **0** | **0** |
| **UGT4** | **1** | **0** | **0** | **0** | **0** | **0** | **0** | **0** | **0** | **0** | **0** | **0** | **0** | **0** | **0** | **0** | **0** | **1** | **0** | **0** | **1** | **0** |
| **UGT5** | **0** | **0** | **1** | **0** | **0** | **0** | **0** | **0** | **0** | **0** | **0** | **0** | **0** | **0** | **0** | **0** | **0** | **0** | **0** | **0** | **0** | **0** |
| **UGT6** | **0** | **0** | **0** | **1** | **0** | **0** | **0** | **0** | **0** | **0** | **0** | **0** | **0** | **0** | **1** | **0** | **0** | **0** | **0** | **0** | **0** | **0** |
| **UGT7** | **0** | **0** | **0** | **0** | **0** | **0** | **0** | **0** | **0** | **0** | **1** | **0** | **0** | **0** | **0** | **0** | **0** | **0** | **0** | **0** | **0** | **0** |
| **UGT8** | **0** | **0** | **0** | **0** | **0** | **0** | **0** | **0** | **0** | **1** | **0** | **0** | **0** | **0** | **0** | **0** | **0** | **0** | **0** | **0** | **0** | **0** |
| **UGT9** | **0** | **0** | **0** | **0** | **0** | **0** | **0** | **0** | **0** | **0** | **0** | **0** | **0** | **1** | **0** | **0** | **0** | **0** | **0** | **1** | **0** | **0** |
| **UGT10** | **0** | **0** | **0** | **0** | **0** | **0** | **0** | **0** | **0** | **1** | **0** | **0** | **0** | **0** | **0** | **0** | **0** | **0** | **0** | **0** | **0** | **0** |
| **UGT11** | **0** | **0** | **1** | **0** | **0** | **0** | **0** | **0** | **0** | **0** | **0** | **0** | **0** | **0** | **0** | **0** | **0** | **0** | **0** | **1** | **0** | **0** |
| **UGT12** | **6** | **4** | **7** | **5** | **4** | **5** | **4** | **6** | **6** | **0** | **5** | **3** | **4** | **4** | **5** | **5** | **5** | **4** | **5** | **3** | **4** | **4** |
| **UGT13** | **1** | **1** | **1** | **1** | **0** | **0** | **0** | **0** | **0** | **0** | **0** | **0** | **0** | **0** | **0** | **0** | **0** | **0** | **0** | **0** | **0** | **0** |
| **UGT14** | **2** | **2** | **2** | **1** | **3** | **3** | **1** | **3** | **5** | **2** | **2** | **3** | **2** | **2** | **2** | **3** | **4** | **3** | **3** | **4** | **3** | **4** |
| **UGT15** | **0** | **0** | **0** | **0** | **0** | **0** | **0** | **0** | **0** | **0** | **0** | **0** | **1** | **0** | **0** | **0** | **0** | **0** | **0** | **0** | **0** | **1** |
| **UGT16** | **0** | **0** | **0** | **0** | **0** | **0** | **0** | **0** | **0** | **0** | **0** | **0** | **0** | **0** | **0** | **0** | **0** | **1** | **0** | **0** | **0** | **0** |
| **UGT17** | **0** | **1** | **1** | **0** | **0** | **0** | **0** | **0** | **0** | **0** | **0** | **0** | **0** | **0** | **0** | **0** | **0** | **0** | **0** | **0** | **0** | **1** |
| **UGT18** | **0** | **0** | **0** | **0** | **0** | **0** | **0** | **0** | **0** | **0** | **0** | **3** | **0** | **0** | **0** | **0** | **0** | **0** | **0** | **0** | **0** | **0** |
| **UGT19** | **2** | **3** | **2** | **4** | **2** | **4** | **0** | **3** | **3** | **0** | **4** | **2** | **2** | **4** | **5** | **0** | **6** | **2** | **4** | **0** | **2** | **2** |
| **UGT20** | **0** | **0** | **0** | **0** | **0** | **0** | **0** | **0** | **0** | **0** | **0** | **0** | **1** | **0** | **0** | **1** | **0** | **0** | **0** | **0** | **0** | **0** |
| **UGT21** | **3** | **2** | **3** | **4** | **4** | **3** | **2** | **4** | **3** | **3** | **2** | **3** | **4** | **3** | **1** | **6** | **2** | **3** | **4** | **3** | **2** | **1** |
| **UGT22** | **4** | **4** | **4** | **3** | **3** | **2** | **4** | **6** | **3** | **0** | **3** | **3** | **5** | **2** | **4** | **2** | **5** | **1** | **3** | **3** | **6** | **3** |
| **UGT23** | **0** | **0** | **0** | **0** | **2** | **0** | **0** | **0** | **0** | **0** | **1** | **0** | **0** | **0** | **0** | **0** | **0** | **0** | **0** | **0** | **0** | **1** |
| **UGT24** | **2** | **2** | **2** | **4** | **1** | **6** | **1** | **3** | **3** | **6** | **2** | **4** | **3** | **4** | **3** | **3** | **4** | **4** | **4** | **1** | **2** | **3** |
| **UGT25** | **1** | **1** | **2** | **2** | **1** | **2** | **2** | **1** | **1** | **2** | **2** | **1** | **2** | **1** | **2** | **1** | **1** | **3** | **2** | **2** | **0** | **1** |
| **UGT26** | **2** | **2** | **1** | **1** | **2** | **1** | **1** | **0** | **1** | **0** | **1** | **1** | **3** | **0** | **2** | **0** | **2** | **3** | **1** | **1** | **2** | **3** |
| **UGT27** | **1** | **0** | **4** | **2** | **3** | **2** | **2** | **4** | **2** | **0** | **3** | **1** | **2** | **3** | **3** | **5** | **5** | **1** | **3** | **2** | **3** | **4** |
| **UGT28** | **3** | **3** | **2** | **3** | **3** | **3** | **2** | **3** | **3** | **2** | **3** | **2** | **4** | **1** | **2** | **2** | **3** | **2** | **2** | **3** | **1** | **3** |
| **UGT29** | **3** | **2** | **3** | **0** | **2** | **3** | **2** | **2** | **2** | **1** | **3** | **2** | **1** | **3** | **3** | **0** | **3** | **1** | **1** | **1** | **3** | **1** |
| **UGT30** | **2** | **2** | **2** | **2** | **1** | **1** | **2** | **3** | **3** | **4** | **2** | **3** | **2** | **1** | **2** | **1** | **1** | **4** | **2** | **2** | **3** | **1** |
| **UGT31** | **0** | **0** | **0** | **0** | **0** | **0** | **0** | **0** | **0** | **0** | **0** | **0** | **0** | **0** | **0** | **0** | **0** | **0** | **1** | **0** | **0** | **0** |
| **UGT32** | **1** | **1** | **2** | **0** | **4** | **2** | **1** | **3** | **1** | **0** | **1** | **3** | **6** | **1** | **2** | **4** | **2** | **2** | **1** | **1** | **2** | **4** |
| **UGT33** | **2** | **2** | **1** | **2** | **2** | **2** | **2** | **2** | **2** | **0** | **2** | **2** | **2** | **3** | **1** | **1** | **2** | **2** | **2** | **4** | **2** | **3** |
| **UGT34** | **0** | **1** | **1** | **1** | **3** | **1** | **1** | **2** | **2** | **0** | **1** | **0** | **1** | **2** | **2** | **2** | **1** | **0** | **1** | **1** | **2** | **0** |
| **UGT35** | **1** | **3** | **1** | **3** | **1** | **2** | **3** | **3** | **1** | **0** | **1** | **0** | **3** | **3** | **1** | **0** | **1** | **2** | **1** | **1** | **2** | **0** |
| **UGT36** | **2** | **2** | **2** | **2** | **3** | **2** | **2** | **1** | **2** | **0** | **2** | **3** | **2** | **0** | **2** | **1** | **2** | **2** | **2** | **3** | **3** | **2** |
| **UGT37** | **3** | **3** | **2** | **3** | **1** | **0** | **0** | **0** | **0** | **2** | **0** | **0** | **0** | **0** | **3** | **0** | **0** | **3** | **0** | **2** | **1** | **0** |
| **UGT38** | **2** | **2** | **3** | **3** | **2** | **2** | **1** | **1** | **1** | **3** | **3** | **2** | **1** | **1** | **4** | **2** | **2** | **2** | **2** | **1** | **3** | **3** |
| **UGT39** | **0** | **0** | **0** | **0** | **0** | **0** | **0** | **0** | **0** | **0** | **1** | **0** | **0** | **0** | **0** | **0** | **0** | **0** | **0** | **0** | **0** | **0** |
| **UGT40** | **0** | **0** | **0** | **0** | **0** | **0** | **0** | **0** | **0** | **0** | **0** | **0** | **0** | **1** | **0** | **0** | **0** | **0** | **0** | **0** | **0** | **0** |
| **UGT41** | **1** | **0** | **0** | **0** | **0** | **0** | **0** | **0** | **0** | **0** | **0** | **0** | **0** | **0** | **0** | **0** | **0** | **0** | **0** | **0** | **0** | **0** |
| **UGT42** | **2** | **0** | **2** | **1** | **3** | **3** | **2** | **5** | **3** | **1** | **1** | **0** | **3** | **3** | **2** | **2** | **2** | **0** | **0** | **3** | **3** | **3** |
| **UGT43** | **3** | **2** | **3** | **3** | **3** | **2** | **1** | **2** | **1** | **2** | **2** | **2** | **3** | **2** | **2** | **1** | **2** | **2** | **2** | **2** | **2** | **2** |
| **UGT44** | **0** | **1** | **0** | **0** | **0** | **0** | **0** | **0** | **0** | **0** | **0** | **0** | **0** | **0** | **0** | **0** | **0** | **0** | **0** | **0** | **0** | **0** |
| **UGT45** | **0** | **1** | **3** | **2** | **3** | **2** | **1** | **1** | **3** | **2** | **2** | **3** | **0** | **4** | **1** | **1** | **1** | **2** | **2** | **3** | **5** | **2** |
| **UGT46** | **2** | **1** | **3** | **2** | **0** | **2** | **2** | **1** | **2** | **0** | **2** | **2** | **0** | **3** | **3** | **3** | **2** | **1** | **1** | **2** | **3** | **0** |
| **UGT47** | **1** | **1** | **1** | **1** | **0** | **0** | **0** | **0** | **3** | **1** | **1** | **2** | **1** | **0** | **1** | **2** | **0** | **1** | **1** | **1** | **0** | **0** |
| **UGT48** | **1** | **3** | **2** | **3** | **2** | **0** | **2** | **2** | **2** | **2** | **1** | **0** | **3** | **1** | **1** | **1** | **1** | **2** | **1** | **3** | **3** | **2** |
| **UGT49** | **2** | **1** | **2** | **2** | **2** | **2** | **2** | **2** | **2** | **2** | **1** | **3** | **1** | **2** | **2** | **1** | **2** | **2** | **2** | **1** | **2** | **1** |
| **UGT50** | **0** | **0** | **0** | **0** | **0** | **0** | **0** | **0** | **0** | **0** | **0** | **1** | **0** | **0** | **0** | **0** | **0** | **0** | **0** | **0** | **0** | **0** |
| **UGT51** | **1** | **2** | **2** | **1** | **1** | **5** | **0** | **1** | **3** | **0** | **1** | **2** | **2** | **1** | **1** | **2** | **1** | **2** | **2** | **1** | **2** | **2** |
| **UGT52** | **1** | **2** | **2** | **2** | **1** | **1** | **0** | **4** | **2** | **0** | **2** | **1** | **2** | **3** | **2** | **2** | **2** | **2** | **2** | **2** | **2** | **2** |
| **UGT53** | **2** | **1** | **2** | **1** | **2** | **2** | **2** | **2** | **0** | **1** | **1** | **2** | **0** | **2** | **2** | **2** | **2** | **2** | **1** | **1** | **2** | **2** |
| **UGT54** | **2** | **0** | **2** | **1** | **1** | **1** | **1** | **1** | **0** | **0** | **1** | **1** | **3** | **2** | **2** | **3** | **1** | **1** | **2** | **3** | **3** | **3** |
| **UGT55** | **0** | **2** | **0** | **3** | **1** | **0** | **0** | **0** | **0** | **2** | **0** | **0** | **0** | **0** | **1** | **2** | **0** | **2** | **0** | **1** | **0** | **0** |
| **UGT56** | **2** | **2** | **1** | **2** | **2** | **4** | **0** | **2** | **1** | **2** | **0** | **1** | **2** | **1** | **3** | **2** | **3** | **2** | **1** | **1** | **1** | **1** |
| **UGT57** | **0** | **0** | **0** | **0** | **0** | **0** | **0** | **0** | **0** | **0** | **0** | **0** | **0** | **0** | **1** | **0** | **0** | **0** | **0** | **0** | **0** | **0** |
| **UGT58** | **1** | **1** | **1** | **1** | **1** | **1** | **1** | **1** | **1** | **0** | **1** | **0** | **2** | **2** | **1** | **1** | **2** | **1** | **1** | **1** | **1** | **1** |
| **UGT59** | **1** | **1** | **1** | **1** | **2** | **1** | **1** | **1** | **1** | **0** | **1** | **1** | **1** | **1** | **1** | **0** | **0** | **1** | **1** | **1** | **1** | **1** |
| **UGT60** | **2** | **2** | **2** | **0** | **2** | **1** | **2** | **2** | **2** | **2** | **1** | **2** | **2** | **2** | **2** | **1** | **2** | **2** | **0** | **2** | **1** | **2** |
| **UGT61** | **1** | **2** | **1** | **1** | **1** | **2** | **1** | **1** | **1** | **0** | **1** | **1** | **2** | **2** | **2** | **2** | **1** | **1** | **1** | **0** | **1** | **2** |
| **UGT62** | **1** | **0** | **0** | **0** | **0** | **0** | **0** | **0** | **0** | **0** | **0** | **0** | **0** | **0** | **1** | **0** | **0** | **1** | **0** | **0** | **0** | **1** |
| **UGT63** | **0** | **3** | **3** | **0** | **2** | **2** | **2** | **2** | **2** | **0** | **0** | **1** | **1** | **1** | **1** | **2** | **2** | **0** | **3** | **0** | **2** | **1** |
| **UGT64** | **0** | **0** | **0** | **0** | **0** | **0** | **0** | **0** | **0** | **0** | **0** | **0** | **0** | **0** | **1** | **0** | **0** | **0** | **0** | **0** | **0** | **0** |
| **UGT65** | **2** | **1** | **1** | **0** | **0** | **2** | **1** | **2** | **2** | **1** | **0** | **1** | **1** | **0** | **2** | **1** | **3** | **1** | **1** | **1** | **2** | **1** |
| **UGT66** | **0** | **0** | **0** | **0** | **1** | **1** | **1** | **0** | **0** | **0** | **1** | **0** | **0** | **1** | **0** | **0** | **1** | **0** | **1** | **0** | **1** | **0** |
| **UGT67** | **0** | **1** | **2** | **1** | **2** | **2** | **1** | **3** | **1** | **0** | **0** | **1** | **0** | **2** | **1** | **0** | **1** | **1** | **1** | **1** | **2** | **1** |
| **UGT68** | **0** | **0** | **1** | **0** | **0** | **0** | **0** | **0** | **0** | **0** | **1** | **0** | **0** | **0** | **0** | **0** | **0** | **0** | **0** | **0** | **0** | **0** |
| **UGT69** | **0** | **1** | **2** | **0** | **1** | **0** | **0** | **1** | **2** | **0** | **0** | **1** | **0** | **1** | **0** | **0** | **1** | **1** | **1** | **1** | **0** | **0** |
| **UGT70** | **0** | **3** | **1** | **0** | **1** | **0** | **0** | **1** | **1** | **1** | **3** | **0** | **0** | **1** | **4** | **0** | **1** | **1** | **1** | **1** | **0** | **2** |
| **UGT71** | **1** | **1** | **1** | **2** | **1** | **2** | **1** | **1** | **1** | **0** | **1** | **1** | **1** | **1** | **2** | **0** | **2** | **1** | **1** | **1** | **2** | **1** |
| **UGT72** | **1** | **1** | **1** | **1** | **1** | **1** | **0** | **2** | **2** | **2** | **0** | **1** | **1** | **0** | **0** | **0** | **1** | **0** | **1** | **0** | **0** | **0** |
| **UGT73** | **1** | **3** | **1** | **2** | **1** | **1** | **0** | **1** | **1** | **0** | **1** | **1** | **0** | **1** | **3** | **0** | **1** | **2** | **2** | **0** | **1** | **1** |
| **UGT74** | **0** | **0** | **0** | **0** | **0** | **0** | **0** | **0** | **0** | **0** | **0** | **0** | **1** | **0** | **0** | **0** | **0** | **0** | **0** | **1** | **2** | **0** |
| **UGT75** | **0** | **0** | **0** | **0** | **0** | **0** | **0** | **0** | **1** | **0** | **1** | **0** | **1** | **0** | **0** | **0** | **0** | **0** | **0** | **0** | **0** | **0** |
| **UGT76** | **0** | **0** | **1** | **1** | **1** | **1** | **1** | **1** | **0** | **0** | **0** | **0** | **1** | **0** | **0** | **0** | **1** | **0** | **0** | **1** | **1** | **1** |
| **UGT77** | **0** | **0** | **0** | **0** | **0** | **0** | **1** | **0** | **1** | **0** | **0** | **0** | **1** | **0** | **2** | **0** | **0** | **0** | **0** | **0** | **0** | **1** |
| **UGT78** | **1** | **1** | **1** | **1** | **2** | **1** | **1** | **1** | **1** | **1** | **1** | **2** | **1** | **0** | **1** | **1** | **1** | **0** | **1** | **1** | **2** | **1** |
| **UGT79** | **1** | **1** | **1** | **1** | **0** | **1** | **1** | **1** | **1** | **0** | **1** | **1** | **0** | **1** | **1** | **2** | **2** | **1** | **1** | **1** | **2** | **1** |
| **UGT80** | **0** | **0** | **0** | **0** | **0** | **1** | **0** | **0** | **0** | **0** | **0** | **0** | **0** | **0** | **0** | **0** | **0** | **0** | **0** | **0** | **0** | **0** |
| **UGT81** | **1** | **1** | **1** | **1** | **1** | **1** | **1** | **1** | **1** | **1** | **1** | **1** | **1** | **1** | **1** | **1** | **1** | **1** | **1** | **1** | **2** | **1** |
| **UGT82** | **0** | **0** | **1** | **0** | **0** | **0** | **0** | **0** | **0** | **0** | **0** | **0** | **0** | **0** | **0** | **1** | **0** | **0** | **0** | **0** | **0** | **0** |
| **UGT83** | **0** | **0** | **0** | **0** | **0** | **1** | **0** | **0** | **0** | **0** | **0** | **1** | **1** | **0** | **1** | **0** | **0** | **1** | **0** | **0** | **0** | **0** |
| **UGT84** | **0** | **1** | **2** | **1** | **1** | **2** | **0** | **1** | **1** | **1** | **1** | **1** | **1** | **0** | **0** | **0** | **0** | **1** | **0** | **0** | **1** | **1** |
| **UGT85** | **0** | **0** | **0** | **0** | **0** | **0** | **1** | **0** | **0** | **0** | **0** | **0** | **0** | **0** | **0** | **0** | **0** | **1** | **0** | **0** | **0** | **0** |
| **UGT86** | **0** | **0** | **1** | **1** | **0** | **0** | **0** | **0** | **0** | **0** | **0** | **1** | **1** | **0** | **1** | **1** | **1** | **0** | **1** | **1** | **1** | **1** |
| **UGT87** | **1** | **0** | **1** | **0** | **0** | **0** | **1** | **0** | **0** | **0** | **1** | **0** | **0** | **0** | **1** | **1** | **1** | **1** | **1** | **0** | **1** | **0** |
| **UGT88** | **1** | **0** | **1** | **0** | **1** | **0** | **0** | **0** | **0** | **0** | **0** | **0** | **0** | **0** | **1** | **0** | **0** | **0** | **0** | **0** | **0** | **0** |
| **UGT89** | **0** | **0** | **0** | **0** | **0** | **0** | **1** | **0** | **0** | **0** | **0** | **0** | **0** | **0** | **0** | **0** | **0** | **0** | **0** | **0** | **0** | **0** |
| **UGT90** | **1** | **1** | **1** | **1** | **0** | **1** | **1** | **1** | **1** | **1** | **1** | **0** | **1** | **1** | **1** | **1** | **1** | **1** | **1** | **1** | **1** | **1** |
| **UGT91** | **0** | **1** | **1** | **0** | **0** | **0** | **0** | **0** | **0** | **0** | **0** | **1** | **0** | **0** | **0** | **0** | **0** | **0** | **0** | **0** | **1** | **0** |
| **UGT92** | **0** | **0** | **0** | **0** | **0** | **0** | **0** | **0** | **1** | **0** | **0** | **0** | **0** | **0** | **0** | **0** | **0** | **0** | **0** | **0** | **0** | **0** |
| **UGT93** | **0** | **0** | **0** | **0** | **0** | **0** | **0** | **0** | **0** | **0** | **0** | **0** | **0** | **0** | **0** | **0** | **0** | **0** | **0** | **0** | **0** | **1** |
| **UGT94** | **0** | **1** | **1** | **0** | **1** | **0** | **1** | **1** | **0** | **1** | **0** | **1** | **0** | **1** | **1** | **1** | **0** | **0** | **1** | **1** | **1** | **0** |
| **UGT95** | **0** | **0** | **0** | **0** | **0** | **0** | **0** | **0** | **0** | **0** | **0** | **0** | **0** | **0** | **0** | **0** | **0** | **0** | **0** | **1** | **0** | **0** |
| **UGT96** | **0** | **1** | **1** | **0** | **1** | **0** | **0** | **1** | **0** | **1** | **0** | **0** | **0** | **0** | **1** | **0** | **1** | **1** | **1** | **1** | **1** | **0** |
| **UGT97** | **1** | **1** | **1** | **1** | **1** | **1** | **1** | **1** | **1** | **0** | **1** | **1** | **1** | **1** | **1** | **1** | **1** | **1** | **1** | **2** | **1** | **1** |
| **UGT98** | **1** | **0** | **1** | **1** | **1** | **1** | **1** | **1** | **1** | **1** | **1** | **2** | **1** | **1** | **0** | **1** | **1** | **1** | **2** | **1** | **1** | **1** |
| **UGT99** | **0** | **0** | **0** | **0** | **0** | **0** | **0** | **0** | **0** | **0** | **0** | **0** | **0** | **0** | **0** | **0** | **0** | **0** | **0** | **0** | **1** | **0** |
| **UGT100** | **0** | **1** | **1** | **1** | **2** | **1** | **0** | **1** | **1** | **1** | **2** | **0** | **1** | **0** | **1** | **0** | **1** | **1** | **2** | **1** | **1** | **0** |
| **UGT101** | **0** | **1** | **0** | **0** | **0** | **0** | **0** | **0** | **1** | **0** | **0** | **0** | **0** | **0** | **0** | **0** | **0** | **0** | **0** | **0** | **0** | **0** |
| **UGT102** | **0** | **0** | **1** | **0** | **0** | **0** | **0** | **0** | **0** | **0** | **0** | **0** | **0** | **0** | **0** | **0** | **0** | **0** | **0** | **0** | **0** | **0** |
| **UGT103** | **0** | **0** | **0** | **0** | **0** | **0** | **0** | **0** | **0** | **0** | **0** | **0** | **0** | **0** | **1** | **0** | **0** | **0** | **0** | **0** | **0** | **0** |
| **UGT104** | **1** | **1** | **1** | **1** | **0** | **0** | **0** | **0** | **0** | **1** | **1** | **0** | **1** | **1** | **0** | **1** | **1** | **1** | **1** | **1** | **1** | **1** |
| **UGT105** | **1** | **0** | **1** | **1** | **1** | **2** | **0** | **2** | **1** | **0** | **1** | **1** | **1** | **1** | **1** | **1** | **1** | **1** | **1** | **1** | **1** | **1** |
| **UGT106** | **0** | **1** | **0** | **0** | **1** | **1** | **0** | **1** | **1** | **0** | **0** | **1** | **1** | **1** | **1** | **0** | **1** | **1** | **1** | **1** | **1** | **1** |
| **UGT107** | **1** | **1** | **1** | **0** | **1** | **1** | **2** | **1** | **0** | **1** | **1** | **0** | **1** | **1** | **1** | **1** | **1** | **1** | **1** | **1** | **1** | **1** |
| **UGT108** | **1** | **1** | **1** | **1** | **1** | **0** | **1** | **1** | **1** | **1** | **1** | **1** | **1** | **1** | **1** | **0** | **1** | **0** | **1** | **1** | **1** | **0** |
| **UGT109** | **1** | **1** | **1** | **0** | **1** | **1** | **1** | **1** | **1** | **0** | **1** | **1** | **1** | **1** | **1** | **0** | **1** | **1** | **1** | **1** | **1** | **1** |
| **UGT110** | **1** | **1** | **2** | **1** | **1** | **1** | **1** | **1** | **0** | **2** | **0** | **0** | **0** | **1** | **1** | **1** | **1** | **1** | **1** | **1** | **1** | **1** |
| **UGT111** | **1** | **1** | **1** | **1** | **1** | **1** | **0** | **1** | **1** | **0** | **1** | **0** | **1** | **1** | **1** | **1** | **1** | **1** | **0** | **1** | **1** | **1** |
| **UGT112** | **1** | **1** | **1** | **0** | **1** | **1** | **1** | **1** | **1** | **0** | **1** | **0** | **1** | **1** | **1** | **0** | **1** | **1** | **1** | **1** | **1** | **1** |
| **UGT113** | **1** | **1** | **1** | **0** | **1** | **1** | **0** | **0** | **1** | **1** | **1** | **1** | **1** | **1** | **1** | **1** | **1** | **1** | **1** | **0** | **0** | **1** |
| **UGT114** | **1** | **1** | **1** | **1** | **1** | **1** | **1** | **1** | **1** | **1** | **0** | **1** | **0** | **1** | **1** | **1** | **1** | **1** | **1** | **1** | **1** | **1** |
| **UGT115** | **1** | **0** | **1** | **1** | **0** | **1** | **0** | **1** | **1** | **0** | **1** | **0** | **1** | **1** | **1** | **1** | **0** | **1** | **1** | **1** | **1** | **2** |
| **UGT116** | **1** | **1** | **1** | **1** | **0** | **0** | **1** | **2** | **1** | **0** | **0** | **1** | **1** | **1** | **1** | **2** | **1** | **1** | **1** | **1** | **0** | **1** |
| **UGT117** | **1** | **1** | **1** | **1** | **1** | **1** | **1** | **1** | **1** | **0** | **1** | **1** | **0** | **0** | **1** | **1** | **1** | **1** | **1** | **1** | **1** | **1** |
| **UGT118** | **1** | **1** | **1** | **1** | **1** | **1** | **1** | **1** | **1** | **0** | **0** | **0** | **0** | **1** | **1** | **1** | **1** | **1** | **1** | **1** | **1** | **0** |
| **UGT119** | **2** | **1** | **2** | **0** | **0** | **1** | **1** | **2** | **0** | **0** | **1** | **0** | **1** | **1** | **1** | **1** | **0** | **2** | **1** | **0** | **1** | **0** |
| **UGT120** | **1** | **1** | **1** | **1** | **0** | **1** | **1** | **1** | **1** | **1** | **1** | **0** | **1** | **1** | **1** | **0** | **1** | **1** | **1** | **1** | **1** | **1** |
| **UGT121** | **1** | **1** | **1** | **1** | **1** | **1** | **1** | **1** | **1** | **1** | **1** | **1** | **1** | **1** | **0** | **1** | **0** | **1** | **1** | **0** | **1** | **1** |
| **UGT122** | **1** | **1** | **1** | **1** | **1** | **1** | **1** | **0** | **1** | **1** | **0** | **1** | **0** | **1** | **0** | **1** | **1** | **1** | **1** | **1** | **1** | **1** |
| **UGT123** | **1** | **1** | **1** | **0** | **1** | **1** | **1** | **1** | **1** | **0** | **0** | **1** | **0** | **1** | **1** | **0** | **1** | **1** | **1** | **1** | **2** | **0** |
| **UGT124** | **0** | **0** | **0** | **0** | **0** | **1** | **0** | **0** | **0** | **0** | **0** | **0** | **0** | **0** | **0** | **0** | **0** | **0** | **0** | **0** | **0** | **0** |
| **UGT125** | **0** | **0** | **0** | **1** | **0** | **0** | **0** | **0** | **0** | **0** | **0** | **0** | **0** | **0** | **1** | **0** | **0** | **0** | **0** | **0** | **0** | **0** |
| **UGT126** | **0** | **0** | **1** | **0** | **1** | **0** | **1** | **0** | **0** | **1** | **0** | **1** | **0** | **1** | **0** | **0** | **0** | **0** | **2** | **0** | **0** | **0** |
| **UGT127** | **1** | **1** | **1** | **1** | **1** | **0** | **1** | **1** | **1** | **0** | **1** | **1** | **1** | **1** | **1** | **0** | **1** | **1** | **1** | **0** | **1** | **1** |
| **UGT128** | **0** | **0** | **0** | **0** | **0** | **0** | **0** | **0** | **0** | **0** | **0** | **0** | **0** | **0** | **0** | **0** | **0** | **0** | **0** | **1** | **0** | **0** |
| **UGT129** | **1** | **1** | **1** | **0** | **0** | **0** | **1** | **0** | **0** | **0** | **0** | **0** | **0** | **0** | **0** | **1** | **0** | **0** | **0** | **0** | **0** | **0** |
| **UGT130** | **1** | **1** | **1** | **1** | **0** | **1** | **0** | **1** | **1** | **1** | **0** | **0** | **1** | **1** | **0** | **1** | **1** | **1** | **1** | **1** | **0** | **1** |
| **UGT131** | **0** | **0** | **1** | **0** | **0** | **0** | **0** | **0** | **0** | **0** | **0** | **0** | **0** | **0** | **0** | **0** | **0** | **0** | **0** | **0** | **0** | **1** |
| **UGT132** | **1** | **0** | **1** | **0** | **0** | **1** | **1** | **1** | **1** | **0** | **1** | **0** | **1** | **1** | **0** | **1** | **1** | **1** | **1** | **0** | **0** | **0** |
| **UGT133** | **1** | **1** | **1** | **0** | **0** | **1** | **0** | **1** | **1** | **0** | **0** | **0** | **1** | **0** | **1** | **1** | **1** | **1** | **1** | **2** | **1** | **1** |
| **UGT134** | **1** | **1** | **0** | **1** | **0** | **1** | **0** | **0** | **0** | **1** | **1** | **1** | **1** | **1** | **1** | **0** | **0** | **1** | **1** | **0** | **0** | **1** |
| **UGT135** | **0** | **0** | **1** | **0** | **0** | **0** | **0** | **0** | **0** | **0** | **0** | **0** | **0** | **0** | **0** | **0** | **0** | **0** | **0** | **0** | **0** | **0** |
| **UGT136** | **0** | **0** | **1** | **0** | **1** | **0** | **0** | **0** | **0** | **0** | **1** | **0** | **0** | **0** | **1** | **0** | **0** | **1** | **0** | **0** | **1** | **1** |
| **UGT137** | **0** | **0** | **0** | **0** | **1** | **0** | **0** | **0** | **0** | **0** | **0** | **0** | **1** | **0** | **0** | **0** | **0** | **0** | **0** | **1** | **0** | **1** |
| **UGT138** | **0** | **0** | **0** | **0** | **0** | **0** | **0** | **0** | **1** | **0** | **0** | **0** | **0** | **0** | **0** | **0** | **0** | **0** | **0** | **0** | **0** | **0** |
| **UGT139** | **1** | **0** | **1** | **0** | **0** | **0** | **1** | **0** | **1** | **1** | **0** | **0** | **0** | **0** | **0** | **0** | **0** | **0** | **0** | **0** | **0** | **0** |
| **UGT140** | **0** | **0** | **0** | **0** | **0** | **0** | **0** | **0** | **0** | **0** | **0** | **0** | **1** | **1** | **0** | **0** | **0** | **2** | **0** | **1** | **0** | **0** |
| **UGT141** | **1** | **0** | **0** | **0** | **0** | **0** | **0** | **1** | **1** | **0** | **0** | **0** | **1** | **1** | **2** | **0** | **1** | **1** | **1** | **0** | **0** | **1** |
| **UGT142** | **1** | **0** | **1** | **1** | **0** | **0** | **0** | **0** | **0** | **0** | **2** | **1** | **1** | **0** | **0** | **0** | **0** | **0** | **0** | **0** | **1** | **0** |
| **UGT143** | **0** | **0** | **1** | **0** | **0** | **0** | **0** | **0** | **0** | **0** | **0** | **0** | **0** | **0** | **0** | **0** | **0** | **0** | **0** | **0** | **0** | **0** |
| **UGT144** | **1** | **0** | **0** | **0** | **1** | **0** | **0** | **0** | **0** | **0** | **0** | **0** | **0** | **0** | **0** | **0** | **0** | **0** | **0** | **0** | **1** | **0** |
| **UGT145** | **1** | **0** | **0** | **0** | **0** | **0** | **0** | **0** | **1** | **0** | **0** | **0** | **0** | **0** | **1** | **1** | **1** | **1** | **0** | **0** | **0** | **1** |
| **UGT146** | **0** | **0** | **0** | **0** | **1** | **0** | **0** | **1** | **0** | **0** | **0** | **1** | **0** | **0** | **0** | **0** | **0** | **0** | **0** | **0** | **0** | **0** |
| **UGT147** | **1** | **0** | **0** | **0** | **1** | **0** | **0** | **1** | **1** | **1** | **0** | **0** | **0** | **1** | **1** | **0** | **0** | **0** | **0** | **0** | **1** | **1** |
| **UGT148** | **1** | **0** | **1** | **0** | **0** | **0** | **0** | **0** | **0** | **0** | **2** | **0** | **1** | **0** | **1** | **0** | **0** | **0** | **0** | **0** | **1** | **1** |
| **UGT149** | **1** | **0** | **0** | **0** | **0** | **0** | **1** | **1** | **0** | **0** | **0** | **0** | **0** | **0** | **0** | **0** | **0** | **0** | **0** | **2** | **0** | **0** |
| **UGT150** | **0** | **0** | **0** | **0** | **0** | **0** | **0** | **1** | **0** | **0** | **1** | **0** | **0** | **0** | **0** | **0** | **1** | **0** | **1** | **0** | **0** | **1** |
| **UGT151** | **0** | **0** | **0** | **0** | **0** | **0** | **0** | **0** | **0** | **0** | **0** | **1** | **0** | **0** | **0** | **0** | **0** | **0** | **0** | **0** | **0** | **0** |
| **UGT152** | **1** | **0** | **0** | **0** | **0** | **0** | **1** | **1** | **0** | **0** | **1** | **0** | **0** | **0** | **1** | **0** | **0** | **2** | **1** | **0** | **0** | **0** |
| **UGT153** | **0** | **0** | **0** | **0** | **0** | **0** | **0** | **1** | **0** | **1** | **1** | **0** | **0** | **0** | **1** | **0** | **0** | **0** | **1** | **0** | **0** | **1** |
| **UGT154** | **0** | **0** | **0** | **0** | **0** | **0** | **0** | **0** | **0** | **0** | **0** | **0** | **0** | **0** | **1** | **0** | **0** | **0** | **0** | **0** | **0** | **0** |
| **UGT155** | **0** | **0** | **0** | **0** | **0** | **2** | **0** | **0** | **0** | **0** | **1** | **0** | **1** | **1** | **1** | **0** | **1** | **1** | **0** | **0** | **0** | **0** |
| **UGT156** | **0** | **0** | **0** | **0** | **0** | **0** | **0** | **0** | **0** | **0** | **0** | **1** | **0** | **0** | **0** | **0** | **0** | **0** | **1** | **0** | **1** | **0** |
| **UGT157** | **0** | **1** | **0** | **1** | **0** | **0** | **0** | **0** | **0** | **0** | **1** | **0** | **1** | **0** | **0** | **0** | **0** | **0** | **0** | **0** | **1** | **0** |
| **UGT158** | **0** | **0** | **3** | **0** | **0** | **0** | **0** | **0** | **0** | **0** | **0** | **1** | **0** | **0** | **0** | **0** | **0** | **0** | **0** | **0** | **1** | **0** |
| **UGT159** | **0** | **0** | **1** | **0** | **1** | **1** | **0** | **0** | **0** | **0** | **0** | **0** | **0** | **2** | **0** | **0** | **1** | **1** | **0** | **0** | **0** | **0** |
| **UGT160** | **0** | **0** | **0** | **1** | **0** | **0** | **0** | **0** | **0** | **1** | **0** | **0** | **0** | **0** | **1** | **0** | **0** | **1** | **0** | **0** | **0** | **0** |
| **UGT161** | **0** | **1** | **0** | **0** | **0** | **0** | **0** | **0** | **1** | **0** | **0** | **0** | **1** | **0** | **1** | **0** | **0** | **0** | **1** | **0** | **1** | **0** |
| **UGT162** | **2** | **0** | **0** | **0** | **1** | **0** | **1** | **0** | **0** | **0** | **0** | **0** | **0** | **0** | **0** | **0** | **0** | **0** | **0** | **0** | **1** | **0** |
| **UGT163** | **0** | **0** | **0** | **0** | **0** | **0** | **0** | **0** | **0** | **0** | **1** | **0** | **0** | **0** | **0** | **0** | **0** | **0** | **0** | **0** | **0** | **0** |
| **UGT164** | **0** | **0** | **1** | **0** | **0** | **0** | **1** | **1** | **0** | **0** | **0** | **1** | **0** | **1** | **0** | **0** | **0** | **0** | **0** | **0** | **0** | **0** |
| **UGT165** | **0** | **0** | **0** | **0** | **0** | **0** | **0** | **0** | **1** | **0** | **1** | **0** | **0** | **0** | **0** | **0** | **0** | **0** | **1** | **0** | **0** | **1** |
| **UGT166** | **1** | **1** | **0** | **0** | **0** | **0** | **0** | **0** | **0** | **0** | **0** | **0** | **0** | **1** | **1** | **0** | **0** | **0** | **0** | **0** | **0** | **0** |
| **UGT167** | **1** | **0** | **0** | **0** | **0** | **0** | **0** | **0** | **0** | **0** | **0** | **0** | **0** | **1** | **0** | **0** | **0** | **0** | **0** | **0** | **0** | **0** |
| **UGT168** | **0** | **1** | **1** | **0** | **0** | **0** | **0** | **0** | **0** | **0** | **0** | **0** | **0** | **0** | **1** | **0** | **0** | **0** | **0** | **0** | **0** | **1** |
| **UGT169** | **0** | **1** | **0** | **0** | **0** | **0** | **0** | **0** | **0** | **0** | **0** | **0** | **0** | **0** | **0** | **0** | **0** | **0** | **0** | **0** | **0** | **0** |
| **UGT170** | **0** | **0** | **1** | **0** | **0** | **0** | **0** | **0** | **0** | **0** | **0** | **0** | **0** | **0** | **1** | **0** | **0** | **0** | **0** | **0** | **0** | **0** |
| **UGT171** | **0** | **0** | **0** | **0** | **0** | **0** | **2** | **0** | **0** | **0** | **0** | **0** | **0** | **0** | **0** | **0** | **0** | **0** | **0** | **0** | **0** | **0** |
| **UGT172** | **0** | **0** | **0** | **0** | **0** | **0** | **0** | **0** | **0** | **0** | **1** | **0** | **0** | **0** | **1** | **1** | **0** | **0** | **1** | **0** | **0** | **0** |
| **UGT173** | **0** | **0** | **0** | **0** | **0** | **0** | **0** | **0** | **0** | **0** | **0** | **0** | **0** | **0** | **0** | **0** | **0** | **0** | **1** | **0** | **0** | **0** |
| **UGT174** | **1** | **0** | **0** | **0** | **0** | **0** | **0** | **0** | **0** | **0** | **0** | **0** | **0** | **0** | **0** | **0** | **0** | **0** | **0** | **0** | **0** | **0** |
| **UGT175** | **0** | **0** | **0** | **0** | **1** | **0** | **0** | **0** | **0** | **0** | **0** | **1** | **0** | **0** | **0** | **0** | **0** | **1** | **0** | **0** | **0** | **0** |
| **UGT176** | **0** | **0** | **0** | **0** | **0** | **0** | **0** | **0** | **0** | **0** | **0** | **0** | **0** | **0** | **1** | **0** | **0** | **0** | **0** | **0** | **0** | **0** |
| **UGT177** | **0** | **0** | **0** | **0** | **0** | **0** | **1** | **1** | **0** | **0** | **0** | **0** | **0** | **0** | **1** | **0** | **0** | **0** | **0** | **0** | **0** | **0** |
| **UGT178** | **0** | **0** | **0** | **0** | **0** | **0** | **0** | **1** | **1** | **0** | **0** | **0** | **0** | **0** | **0** | **0** | **0** | **0** | **0** | **0** | **0** | **0** |
| **UGT179** | **0** | **0** | **0** | **0** | **0** | **0** | **0** | **0** | **0** | **0** | **0** | **1** | **0** | **1** | **1** | **0** | **0** | **0** | **0** | **0** | **0** | **0** |
| **UGT180** | **0** | **0** | **0** | **0** | **0** | **0** | **0** | **0** | **0** | **0** | **0** | **0** | **0** | **0** | **0** | **0** | **0** | **0** | **0** | **1** | **0** | **0** |
| **UGT181** | **1** | **0** | **0** | **0** | **0** | **0** | **0** | **0** | **0** | **0** | **0** | **0** | **0** | **0** | **0** | **0** | **0** | **0** | **0** | **0** | **0** | **0** |
| **UGT182** | **0** | **0** | **0** | **0** | **1** | **0** | **0** | **0** | **0** | **0** | **0** | **0** | **0** | **0** | **0** | **0** | **0** | **1** | **0** | **0** | **0** | **0** |
| **UGT183** | **0** | **0** | **0** | **0** | **0** | **1** | **0** | **0** | **0** | **0** | **0** | **0** | **0** | **0** | **0** | **0** | **0** | **0** | **0** | **0** | **0** | **1** |
| **UGT184** | **0** | **0** | **0** | **0** | **0** | **1** | **0** | **0** | **0** | **0** | **0** | **0** | **0** | **1** | **0** | **0** | **0** | **0** | **0** | **0** | **0** | **0** |
| **UGT185** | **0** | **0** | **0** | **0** | **0** | **0** | **1** | **0** | **0** | **0** | **0** | **0** | **0** | **0** | **0** | **0** | **0** | **1** | **0** | **0** | **0** | **0** |
| **UGT186** | **0** | **0** | **0** | **0** | **0** | **0** | **0** | **0** | **0** | **0** | **0** | **0** | **0** | **0** | **0** | **0** | **0** | **0** | **1** | **0** | **0** | **0** |
| **UGT187** | **0** | **0** | **0** | **0** | **0** | **0** | **0** | **0** | **1** | **0** | **0** | **0** | **0** | **1** | **0** | **0** | **0** | **0** | **0** | **0** | **0** | **0** |
| **UGT188** | **0** | **0** | **0** | **0** | **0** | **0** | **0** | **0** | **0** | **0** | **0** | **1** | **0** | **0** | **0** | **0** | **0** | **0** | **0** | **0** | **0** | **0** |
| **UGT189** | **0** | **0** | **0** | **0** | **0** | **0** | **0** | **0** | **0** | **0** | **0** | **1** | **0** | **0** | **0** | **0** | **0** | **0** | **0** | **0** | **0** | **0** |
| **UGT190** | **0** | **0** | **0** | **0** | **0** | **0** | **0** | **0** | **0** | **0** | **0** | **0** | **0** | **1** | **1** | **0** | **0** | **0** | **0** | **0** | **0** | **0** |
| **UGT191** | **0** | **0** | **1** | **0** | **0** | **0** | **0** | **0** | **0** | **0** | **0** | **0** | **0** | **0** | **0** | **0** | **0** | **0** | **0** | **0** | **0** | **0** |
| **UGT192** | **0** | **0** | **1** | **0** | **0** | **0** | **0** | **0** | **0** | **0** | **0** | **0** | **0** | **0** | **0** | **0** | **0** | **0** | **0** | **0** | **0** | **0** |
| **UGT193** | **0** | **0** | **0** | **0** | **1** | **0** | **0** | **0** | **0** | **0** | **0** | **0** | **0** | **0** | **0** | **0** | **0** | **0** | **0** | **0** | **0** | **0** |
| **UGT194** | **0** | **0** | **0** | **0** | **0** | **0** | **0** | **0** | **0** | **0** | **1** | **0** | **0** | **0** | **0** | **0** | **0** | **0** | **0** | **0** | **0** | **0** |
| **UGT195** | **0** | **0** | **0** | **0** | **0** | **0** | **0** | **0** | **0** | **0** | **0** | **0** | **0** | **0** | **0** | **1** | **0** | **0** | **0** | **0** | **0** | **0** |
| **UGT196** | **0** | **0** | **0** | **0** | **0** | **0** | **0** | **0** | **0** | **0** | **0** | **0** | **0** | **0** | **0** | **1** | **0** | **0** | **0** | **0** | **0** | **0** |
| **UGT197** | **0** | **0** | **0** | **0** | **0** | **0** | **0** | **0** | **0** | **0** | **0** | **0** | **0** | **0** | **0** | **0** | **0** | **1** | **0** | **0** | **0** | **0** |
| **UGT198** | **0** | **0** | **0** | **0** | **0** | **0** | **0** | **0** | **0** | **0** | **0** | **0** | **0** | **0** | **0** | **0** | **0** | **0** | **0** | **1** | **0** | **0** |
| **UGT199** | **0** | **0** | **0** | **0** | **0** | **0** | **0** | **0** | **0** | **0** | **0** | **0** | **0** | **0** | **0** | **0** | **0** | **0** | **0** | **0** | **1** | **0** |
| **UGT200** | **0** | **0** | **0** | **0** | **0** | **0** | **0** | **0** | **0** | **0** | **0** | **0** | **0** | **0** | **0** | **0** | **0** | **0** | **0** | **0** | **0** | **1** |
| **UGT201** | **0** | **0** | **0** | **0** | **0** | **0** | **0** | **0** | **0** | **0** | **0** | **0** | **0** | **0** | **0** | **0** | **0** | **0** | **0** | **0** | **0** | **1** |

| **Table S2 Number of cis-acting elements in HD** | | |
| --- | --- | --- |
| **number** | **function** | **class** |
| **580** | part of a conserved DNA module involved in light responsiveness | Plant growth and development |
| **490** | part of a light responsive element | Plant growth and development |
| **452** | cis-acting regulatory element involved in the MeJA-responsiveness | Phytohormone responsive |
| **361** | cis-acting regulatory element involved in light responsiveness | Plant growth and development |
| **346** | cis-acting regulatory element essential for the anaerobic induction | Abiotic and biotic stresses |
| **333** | cis-acting element involved in the abscisic acid responsiveness | Phytohormone responsive |
| **214** | light responsive element | Plant growth and development |
| **105** | gibberellin-responsive element | Phytohormone responsive |
| **90** | cis-acting regulatory element involved in zein metabolism regulation | Plant growth and development |
| **90** | cis-acting element involved in low-temperature responsiveness | Abiotic and biotic stresses |
| **83** | cis-acting regulatory element related to meristem expression | Plant growth and development |
| **78** | MYB binding site involved in drought-inducibility | Abiotic and biotic stresses |
| **70** | cis-acting element involved in salicylic acid responsiveness | Phytohormone responsive |
| **70** | cis-acting element involved in defense and stress responsiveness | Abiotic and biotic stresses |
| **69** | auxin-responsive element | Phytohormone responsive |
| **58** | part of a module for light response | Plant growth and development |
| **52** | MYBHv1 binding site | Abiotic and biotic stresses |
| **42** | cis-acting element involved in gibberellin-responsiveness | Phytohormone responsive |
| **39** | part of a light responsive module | Plant growth and development |
| **Table S2 Number of cis-acting elements in TGY** | | |
| **number** | **function** | **class** |
| **515** | part of a conserved DNA module involved in light responsiveness | Plant growth and development |
| **434** | part of a light responsive element | Plant growth and development |
| **404** | cis-acting regulatory element involved in the MeJA-responsiveness | Phytohormone responsive |
| **306** | cis-acting regulatory element involved in light responsiveness | Plant growth and development |
| **302** | cis-acting regulatory element essential for the anaerobic induction | Abiotic and biotic stresses |
| **281** | cis-acting element involved in the abscisic acid responsiveness | Phytohormone responsive |
| **203** | light responsive element | Plant growth and development |
| **97** | gibberellin-responsive element | Phytohormone responsive |
| **88** | cis-acting regulatory element involved in zein metabolism regulation | Plant growth and development |
| **87** | cis-acting regulatory element related to meristem expression | Plant growth and development |
| **71** | cis-acting element involved in low-temperature responsiveness | Abiotic and biotic stresses |
| **69** | auxin-responsive element | Phytohormone responsive |
| **65** | MYB binding site involved in drought-inducibility | Abiotic and biotic stresses |
| **65** | cis-acting element involved in salicylic acid responsiveness | Phytohormone responsive |
| **65** | cis-acting element involved in defense and stress responsiveness | Abiotic and biotic stresses |
| **53** | part of a module for light response | Plant growth and development |
| **48** | MYB binding site involved in light responsiveness | Plant growth and development |
| **38** | cis-acting regulatory element | Plant growth and development |
| **36** | part of a light responsive module | Plant growth and development |
